# Cellular retinoic acid-binding proteins regulate germ cell proliferation and sex determination in zebrafish

**DOI:** 10.1242/dev.202549

**Published:** 2024-12-13

**Authors:** Lianna Fung, Daniel B. Dranow, Arul Subramanian, Natalia Libby, Thomas F. Schilling

**Affiliations:** Department of Developmental and Cell Biology, University of California, Irvine, CA 92697-2300, USA

**Keywords:** Crabp, Germ cell, Gonad development, Retinoic acid, Sex determination, Sex differentiation

## Abstract

Cellular retinoic acid (RA)-binding proteins (Crabps) solubilize intracellular RA and transport it to its nuclear receptors or cytoplasmic degradation enzymes. Despite their extreme conservation across chordates, genetic studies of Crabp function have revealed few essential functions. We have generated loss-of-function mutations in all four zebrafish Crabps and find essential roles for Crabp2 proteins in gonad development and sex determination. Transgenic RA reporters show strong RA responses in germ cells at the bipotential stage of gonad development. Double mutants lacking the functions of both Crabp2a and Crabp2b predominantly become male, which correlates with their smaller gonad size and reduced germ cell proliferation during gonad development at late larval and early juvenile stages. In contrast, mutants lacking the functions of both Crabp1a and Crabp1b have normal sex ratios. Exogenous RA treatments at bipotential gonad stages increase germ cell number, consistent with a direct role for RA in promoting germ cell proliferation*.* Our results suggest essential functions for Crabps in gonad development and sex determination.

## Teaser

**Summary:** Genetic analyses in zebrafish reveal novel roles for cellular retinoic acid binding proteins in germ cells development and sex determination.

## INTRODUCTION

Sex determination is a fundamental feature of animal development that varies greatly across organisms and can involve both genetic and environmental determinants. The processes that regulate sex determination are also surprisingly plastic, even within a species. In zebrafish (*Danio rerio*), wild strains have a ZZ/ZW chromosomal sex-determination system, with ZW animals primarily becoming female and ZZ animals becoming male (Sharma et al., 1998; Wilson et al., 2014). In contrast, laboratory strains (e.g. AB and TU) derived from domesticated animals have lost this chromosomal sex-determination system (Wilson et al., 2014). Although the mechanisms of sex determination in lab strains remain unclear, they are thought to involve contributions from both environmental determinants and multiple genetic loci (Kossack and Draper, 2019).

All-*trans*-retinoic acid (RA) is a cell-cell signaling molecule derived from dietary vitamin A that plays important roles in sex differentiation in many vertebrate species. In mice, RA regulates initiation of meiosis in oocytes, and promotes female sex differentiation through induction of stimulated by RA 8 (*Stra8*), while a RA-degrading enzyme of the cytochrome p450 (Cyp) family prevents *Stra8* expression in embryonic and adult testes (Koubova et al., 2006). RA appears to function differently in zebrafish sex differentiation. Several fish species, including zebrafish, do not have *Stra8* homologues (Pasquier et al., 2016). During the bipotential stage in zebrafish (8-20 days post-fertilization, dpf), most gonad cells (including stromal and follicle cells) express aldehyde dehydrogenase 2 (*aldh1a2*), the primary enzyme that converts retinal to RA, likely generating high levels of RA throughout the tissue (Liu et al., 2022; Rodriguez-Mari et al., 2013). During this period, all zebrafish initially produce early-stage oocytes that mature and continue to be produced in females or are degraded in fish that will become males during sex differentiation (20-25 dpf) (Takahashi, 1977). This process is strongly influenced by the number of germ cells (GCs) present at the bipotential stage, since mutants with complete loss of or reductions in GC number, particularly oocytes, become male (Dranow et al., 2016; Rodriguez-Mari et al., 2010; Siegfried and Nusslein-Volhard, 2008; Slanchev et al., 2005). During sex differentiation, zebrafish express the RA-degrading enzyme *cyp26a1* in a sexually dimorphic manner in the gonad by upregulating somatic expression in males and downregulating expression in females, resulting in low and high RA levels in male and female gonads, respectively (Rodriguez-Mari et al., 2013). Previous studies have focused primarily on RA at these later stages of sex differentiation, both in zebrafish and mammals, but earlier roles for RA in gonad development before oogenesis have not been described.

Cellular retinoic acid-binding proteins (Crabps) bind RA with high affinity and transport it intracellularly (Ong and Chytil, 1978). Vertebrates have two classes of highly conserved Crabps, Crabp1s and Crabp2s, which transport RA to cytochrome p450 enzymes (Cyps, particularly members of the Cyp26 family) for degradation in the cytosol as well as to RA receptors (RARs) in the nucleus (Astrom et al., 1991; Budhu et al., 2001; Budhu and Noy, 2002; Delva et al., 1999). Mammalian CRABP1 and CRABP2 show variable tissue-specific expression, with CRABP2 more commonly expressed in tissues that synthesize RA. CRABP2 transports RA to RARs *in vitro,* as well as to Cyp26s, while CRABP1 primarily transports RA to Cyp26s for degradation (Boylan and Gudas, 1992; Fiorella and Napoli, 1994; Won et al., 2004). Consequently, in general, only elevating CRABP2 levels increases the transcription rate of RA responsive genes, while CRABP1 does not (Napoli, 2017). Zebrafish have four Crabp orthologues, *crabp1a*, *crabp1b*, *crabp2a* and *crabp2b*. Our previous work has shown that, among these orthologues, *crabp2a* is uniquely RA responsive, attenuates noise and promotes robustness in RA levels in the patterning of hindbrain rhombomeres (Cai et al., 2012; Sosnik et al., 2016). Mice lacking both Crabp1 and Crabp2 are viable but have supernumerary forelimb digits at low penetrance (Lampron et al., 1995), suggesting compensation for Crabp functions in RA signaling by other proteins (Romand et al., 2000). Thus, despite their extremely high conservation in all vertebrates, there is little evidence for essential functions *in vivo*.

Here, we show requirements for zebrafish Crabp2s in gonad development and sex determination. Combined loss-of-function of both Crabp2a and Crabp2b leads to a dramatic increase in the proportion of males, which correlates with reduced gonad size, GC proliferation and, ultimately, GC number during early gonad development as well as adulthood. Exogenous RA treatments at bipotential gonad stages promote GC proliferation, suggesting a direct role for RA in early gonad development and sex determination.

## RESULTS AND DISCUSSION

### Crabp2s promote female sex determination

**Fig. 1.**
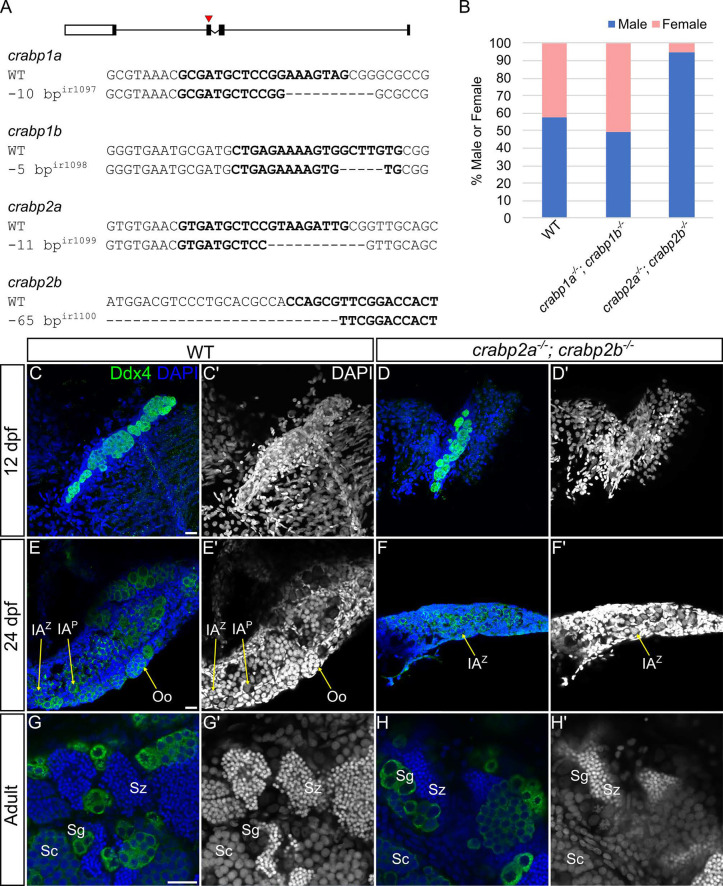
**Crabp2 mutants are disproportionately male and have smaller gonads.** (A) Schematic of the four Crabp1 and Crabp2 genes. Red arrowhead indicates the exon 2 gRNA target site. Selected sequences depict Crabp1 and Crabp2 wild-type gRNA target sites in bold and corresponding mutant alleles for *crabp1a*, *crabp1b*, *crabp2a* and *crabp2b.* (B) Histogram displaying sex ratios in wild types (male, *n*=77; female, *n*=57), *crabp1a^−/−^; crabp1b^−/−^* double mutants (male, *n*=51; female, *n*=52) and *crabp2a^−/−^; crabp2b^−/−^* double mutants (male, *n*=88; female, *n*=5). (C-H′) Representative images of 12 dpf (C-D′), 24 dpf (E-F′) and adult (G-H′) gonads in wild types and *crabp2a^−/−^; crabp2b^−/−^* double mutants. (C-H) Anti-Ddx4 antibody-labeled germ cells (GCs) are green; DAPI-labeled nuclei are blue. (C′-H′) Grayscale DAPI. (C-D′) *Z*-projections; (E-H′) single slices. Oo, oogonia; IA^Z^, zygotene stage IA oocyte; IA^P^, pachytene stage IA oocyte; Sg, spermatogonia; Sc, spermatocytes; Sz, spermatozoa. GC staging according to Draper (2012) and Selman et al. (1993). Scale bars: 20 μm. Wild type: 12 dpf, *n*=4 larvae; 24 dpf, *n*=4 fish; adult, *n*=8 fish. *crabp2a^−/−^; crabp2b^−/−^*mutants: 12 dpf, *n*=4 larvae; 24 dpf, *n*=5 fish; adult, *n*=6 fish.

To investigate requirements for Crabps in zebrafish, we used CRISPR-Cas9-mediated gene editing to generate loss-of-function mutants for all four Crabp genes (Fig. 1A). Upon generation of out-of-frame deletion mutant alleles for each gene, we did not observe any discernible phenotypes in single mutants, either at embryonic or later adult stages. Analyses of RNA levels of other Crabp genes in *crabp2a*^−/−^ or *crabp2b*^−/−^ mutants revealed an almost twofold increase in expression of the remaining Crabp2 paralogue (Fig. S1). We therefore hypothesized that the absence of phenotypes, at least in the case of *crabp2a* and *crabp2b* single mutants, was likely due to genetic compensation.

To address the possibility of genetic compensation by the remaining Crabp paralogues in single mutants, we produced double mutants for *crabp1a*, *crabp1b*, *crabp2a* and *crabp2b*. Both *crabp1a^−/−^; crabp1b^−/−^* and *crabp2a^−/−^; crabp2b^−/−^* double mutants were viable and survived to adulthood with no obvious phenotypes, so we further generated maternal-zygotic (MZ) homozygous double mutants for each, which lack maternally deposited wild-type mRNAs, as well as zygotic transcripts (*crabp1a^−/−^; crabp1b^−/−^* and *crabp2a^−/−^; crabp2b^−/−^*). MZ mutants were also both viable and fertile. Strikingly, however, sex ratios in *crabp2a^−/−^; crabp2b^−/−^* double mutants were dramatically shifted such that nearly all adults appeared to be males (*P*<0.0001, binomial test using expected ratio of 0.5), in contrast to *crabp1a^−/−^; crabp1b^−/−^* double mutants or wild type (*P*=1 and *P*=0.1, binomial tests using expected ratio of 0.5) (Fig. 1B). These data suggested that Crabp2 genes have essential roles in female sex determination and/or differentiation and the maintenance of sex.

To assess possible causes of the skewed sex ratio, we examined gonads at different developmental stages. *crabp2a^−/−^; crabp2b^−/−^* double mutants had notably smaller gonads with fewer GCs, as marked by the GC-specific marker Ddx4 when compared with wild-type animals at the bipotential stage at 12 days postfertilization (dpf) (Fig. 1C-D′). These differences persisted through stages of sex differentiation and into adulthood, although gonads of *crabp2a^−/−^; crabp2b^−/−^* double mutants still appeared to form early oocytes at 24 dpf (Fig. 1E-F′). Adult *crabp2a^−/−^; crabp2b^−/−^* double mutant males had testes that appeared identical to those of wild-type animals (Fig. 1G-H′). This was not surprising as crabp*2a^−/−^; crabp2b^−/−^* double mutants are fertile. The differences in gonad size and GC number that we observed strongly suggest that Crabp2 genes have a role in regulating early gonad development.

### Activation of RA signaling in germ cells of the bipotential gonad

**Fig. 2.**
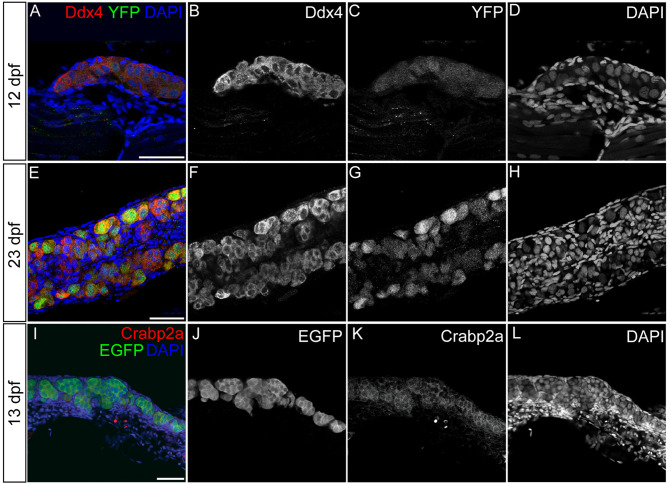
**Germ cells are retinoic acid responsive during early gonad development.** (A-H) Representative confocal images of 12 (A-D) and 23 (E-H) dpf wild-type gonads in *Tg(RARE:YFP)* transgenics. (I-L) Representative confocal images of a 13 dpf wild-type gonad from a *Tg(piwil1:EGFP)* transgenic stained using anti-Crabp2a antibody. (A,E) YFP-labeled RA-responsive cells are green; anti-Ddx4 antibody labeled germ cells (GCs) are red. (I) EGFP-labeled piwil1^+^ GCs are green; Crabp2a-expressing cells are red. (A,E,I) DAPI-labeled nuclei are blue. (B,F) Grayscale Ddx4, (C,G) YFP, (J) EGFP, (K) Crabp2a and (D,H,L) DAPI. (A-D,I-L) *Z*-projections. (E-H) Single slices. Scale bars: 50 μm. Wild type: 12 dpf, *n*=3 larvae; 23 dpf, *n*=1 fish; 13 dpf, *n*=1 fish. Both gonads from each fish were imaged.

As Crabp proteins are intracellular transporters of RA and mediate various aspects of RA signaling, our results suggest that defects in gonad size and GC number in crabp*2a^−/−^; crabp2b^−/−^* double mutants are due to roles for RA signaling in early GC development. However, it remains unclear whether these phenotypes are due to secondary effects from changes in RA signaling in somatic cells or in the GCs themselves. To investigate whether loss of GCs or skewed sex ratios reflect GC autonomous or cell non-autonomous effects, we first looked at RA responses in cells of larval and juvenile zebrafish gonads using a RA-response element (RARE) transgenic reporter line, *Tg(RARE-gata2a:NLS-YFP)^ID1^* [hereafter referred to as *Tg(RARE:YFP)* (Perz-Edwards et al., 2001)]. Interestingly, we observed strong Tg(RARE:YFP) expression specifically in GCs at the early bipotential gonad stage at 12 dpf (Fig. 2A-D). Also consistent with the expression of RA-regulating genes in previous studies (Rodriguez-Mari et al., 2013), elevated Tg(RARE:YFP) expression persisted at least until sex differentiation at 23 dpf (Fig. 2E-H). Using a commercial antibody raised against zebrafish Crabp2a, we found that it was enriched in GCs (Fig. 2I-L). Since the anti-Crabp2a antibody was raised against a proprietary sequence of the protein, to verify its specificity we performed a western blot using protein extract from adult wild type and *crabp2a^−/−^; crabp2b^−/−^*double mutants. This showed drastic reduction (70%) in the Crabp2a protein levels in the mutant compared with wild type (Fig. S2P,Q,U). As Crabp1a, Crabp1b, Crabp2a and Crabp2b proteins share >60% sequence identity, we hypothesize that the antibody cross-reacts with other Crabp members, albeit weakly. Using this antibody, we found that Crabp2a also localized to GCs in adult wild-type testes and testes of *crabp1a^−/−^; crabp1b^−/−^* double mutants, but not in testes of crabp*2a^−/−^; crabp2b^−/−^* double homozygous MZ mutants (Fig. S2A-L). Similarly, *crabp2a* RNA was dramatically reduced in crabp*2a^−/−^; crabp2b^−/−^* double mutants compared to wild type in adult testes (Fig. S2M-O,R-T). These results support a cell-autonomous role for RA signaling in GC development.

### Crabp2 proteins promote germ cell proliferation

Previous studies have shown that higher GC numbers during the bipotential stage in zebrafish favor female sex determination, while reductions or loss of GCs favor male sex determination (Leerberg et al., 2017; Tzung et al., 2015). Notably, laboratory larval zebrafish tend to have a unimodal distribution of GCs in the population at 7 dpf (exhibiting a normal distribution of GCs in a population around a single peak), but by 14 dpf a bimodal shift (exhibiting a distribution of GCs in the population around two peaks) distinguishes larvae with higher GC numbers, which are more likely to develop as females (Tzung et al., 2015). RA promotes proliferation of human GC-like cells and dissected chick primordial GCs in cell culture (Tan et al., 2016; Yu et al., 2012), but this role for RA in promoting GC proliferation has not been tested *in vivo*. We hypothesized that Crabp2s and RA play a role in regulating GC proliferation during early larval stages of zebrafish gonad development.

**Fig. 3.**
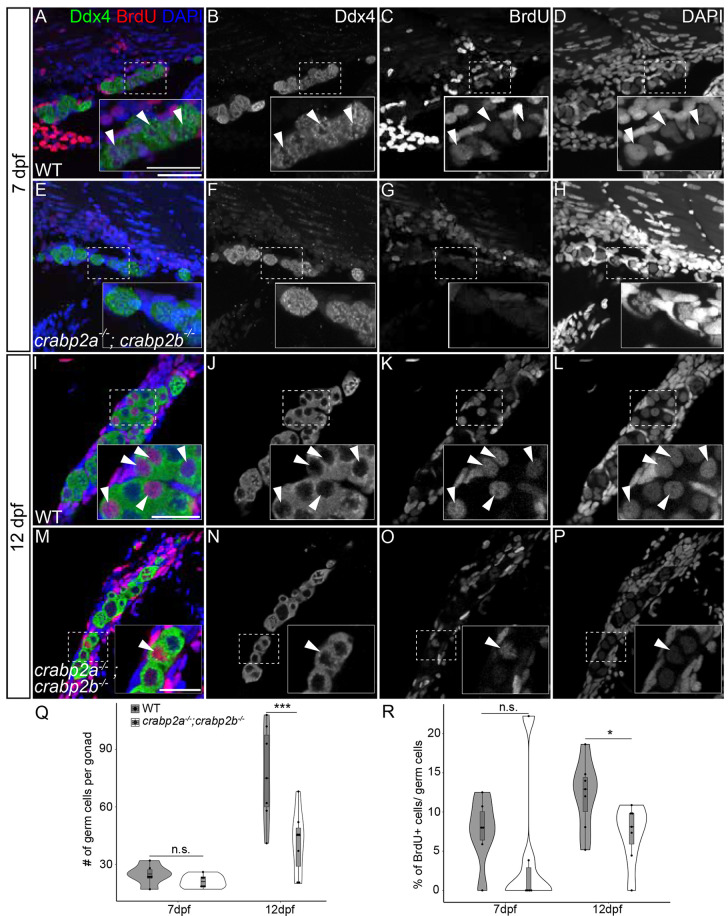
**Crabp2 mutant gonads have fewer germ cells and decreased germ cell proliferation.** (A-P) Representative confocal *z*-projections of BrdU incorporation at 7 (A-H) and 12 (I-P) dpf in gonads of wild type and *crabp2a^−/−^; crabp2b^−/−^* double mutants. (A,E,I,M) Anti-Ddx4 antibody-labeled germ cells (GCs) are green; BrdU-labeled proliferating cell nuclei are red; DAPI-labeled nuclei are blue. (B,F,J,N) Grayscale Ddx4; (C,G,K,O) BrdU; (D,H,L,P) DAPI. Insets show magnified views of regions of interest in GCs (outlined). Arrowheads indicate GC nuclei showing BrdU incorporation. (Q) Violin, and box and whisker plots depicting GC numbers at 7 and 12 dpf. Each datapoint represents total GC number per gonad. At 7 dpf, *crabp2a^−/−^; crabp2b^−/−^* double mutants and wild types are similar (*P*=0.1775). At 12 dpf, *crabp2a^−/−^; crabp2b^−/−^* double mutants have significantly fewer GCs than wild types (*P*=0.0001). (R) Violin, and box and whisker plots depicting the percentages of proliferating GCs at 7 and 12 dpf. Each datapoint represents the number of BrdU^+^ GCs per gonad. At 7 dpf, *crabp2a^−/−^; crabp2b^−/−^* double mutants and wild types are similar (*P*=0.4507). At 12 dpf, *crabp2a^−/−^; crabp2b^−/−^* double mutants have significantly fewer BrdU^+^ GCs compared to wild types (*P*=0.0424). An unpaired two-tailed *t*-test was used to test for significance (*P<0.05; ***P<0.0001; n.s., no significance). Violin plots: wild type, gray bars; *crabp2a^−/−^; crabp2b^−/−^* double mutants, white bars. The box represents the interquartile range (IQR) where 50% of the data points are present. The height of the box is inversely proportional to the clustering of the measurements. Outliers are present outside the box and quartiles. The horizontal line in the box plot represents the median. Scale bars: 50 μm (25 μm in insets). Wild type: 7 dpf, *n*=3 larvae; 12 dpf, *n*=7 fish. *crabp2a^−/−^; crabp2b^−/−^*mutants: 7 dpf, *n*=3 larvae; 12 dpf, *n*=8 fish.

To assess this, we quantified GC numbers in *crabp2a^−/−^; crabp2b^−/−^* double mutant gonads stained with the anti-Ddx4 antibody and compared them to wild-type gonads at the same stages. We observed no significant differences at 7 dpf between *crabp2a^−/−^; crabp2b^−/−^* double mutants and wild type (Fig. 3A-H,Q), but by 12 dpf, *crabp2a^−/−^; crabp2b^−/−^* double mutant gonads had far fewer GCs than wild type gonads (Fig. 3I-Q). Similarly, we observed fewer spermatogonia in adult mutant testes as assessed by counting GCs with distinct nuclear morphology that express the highest levels of Ddx4 (Fig S3A,C,E,G,I,K,M) (Draper, 2023). To assess if this decrease was due to reduced proliferation, we incubated 11 dpf larvae for 24 h in 10 mM BrdU, dissected their gonads at 12 dpf, and counted BrdU and Ddx4 double-positive cells (Fig. 3A,E,I,M). We found that the proportion of BrdU^+^ GCs in *crabp2a^−/−^; crabp2b^−/−^* double mutant gonads was reduced compared to wild-type gonads (Fig. 3R). In contrast, there was no significant difference in GC number or proliferation between wild type and *crabp1a^−/−^; crabp1b^−/−^* double mutants (Figs S4 and S3). We also noticed reduced GC proliferation in adult *crabp2a^−/−^; crabp2b^−/−^* double mutant gonads compared to wild-type gonads, as evidenced by quantification of phospho-histone H3 stained GCs (Fig. S3A,C,D,I,K,L,N). These data indicate that loss of Crabp2 proteins results in decreased GC proliferation early in gonad development, resulting in low GC number during the bipotential stage.

### Novel roles for RA in germ cell proliferation during early gonad development

**Fig. 4.**
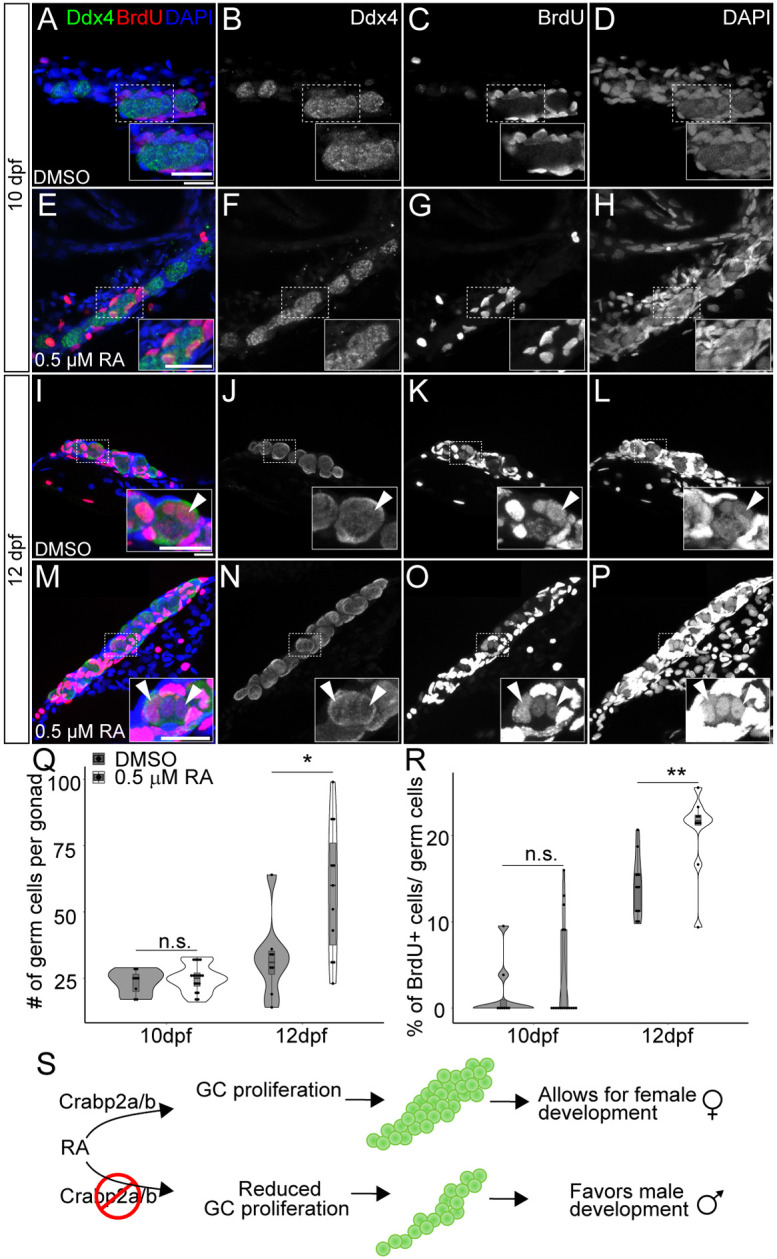
**Retinoic acid promotes germ cell proliferation during gonad development.** (A-H) Representative confocal *z*-projections of BrdU incorporation between 9 and 10 (A-H), and 11 and 12 (I-P) dpf in gonads of DMSO vehicle- (A-D,I-L) or 0.5 µM RA- (E-H,M-P) treated animals. Anti-Ddx4 labeled germ cells (GCs) are green; BrdU-labeled proliferating cell nuclei are red (A,E,I,M). (B,F,J,N) Grayscale Ddx4; (C,G,K,O) BrdU; (D,H,L,P) DAPI. Insets show magnified views of regions of interest in GCs (outlined). Arrowheads indicate GC nuclei showing BrdU incorporation. (Q) Violin, and box and whisker plots depicting the GC number per gonad at 10 and 12 dpf. Each datapoint represents the total GC number in one gonad. At 10 dpf, DMSO- and 0.5 µM RA-treated animals were similar (*P*=0.4930). At 12 dpf, DMSO-treated animals had significantly fewer GCs than 0.5 µM RA-treated animals (*P*=0.0182). (R) Violin, and box and whisker plots depicting percentages of proliferating GCs at 10 and 12 dpf. Each datapoint represents BrdU^+^ GC numbers per gonad. At 10 dpf, most DMSO- and 0.5 µM RA-treated animals showed no BrdU^+^ GCs (*P*=0.4209). At 12 dpf, DMSO-treated animals had significantly fewer BrdU^+^ GCs compared to 0.5 µM RA-treated animals (*P*=0.0044). An unpaired two-tailed *t*-test was used to test for significance (*P<0.05; **P<0.01; n.s., no significance). (S) Schematic for proposed roles of RA and Crabp2 proteins in regulating GC development and sex differentiation. Violin plots: DMSO treatments, gray bars; 0.5 µM RA, white bars. The box represents the interquartile range (IQR) where 50% of the data points are present. The height of the box is inversely proportional to the clustering of the measurements. Outliers are present outside the box and quartiles. The horizontal line in the box plot represents the median. Scale bars: 20 μm. DMSO: 10 dpf, *n*=3 larvae; 12 dpf, *n*=7 fish. RA treated: 10 dpf, *n*=3 larvae; 12 dpf, *n*=8 fish.

Based on these findings, we hypothesized that reduced RA signaling in *crabp2a^−/−^; crabp2b^−/−^* double mutants leads to diminished GC proliferation, suggesting that RA positively regulates GC cell division. To test this, we treated wild-type zebrafish larvae with either 0.5 µM RA or DMSO vehicle alone, starting from 8 dpf. Treated larvae were also incubated in 10 mM BrdU 24 h before their fixation at 10 or 12 dpf to assess proliferation. If RA promotes cell division, we expected to see increased GC numbers and proliferation, as quantified by BrdU incorporation in RA-treated fish. While we did not detect significant differences in GC number or proliferation between DMSO- and RA-treated larvae at 10 dpf, GC number and proliferation increased in 0.5 µM RA-treated larvae at 12 dpf (Fig. 4I-R). Additionally, both DMSO- and RA-treated larvae had reduced numbers of GCs and BrdU incorporation at 12 dpf compared to untreated larvae at the same stages (compare Figs 3Q,R and 4Q,R). This was not surprising since the DMSO- and RA-treated larvae are kept in the dark to prevent RA degradation, and have their treatment medium replaced daily several hours after their daily feeding with live rotifers, reducing both their feeding activity and food availability. These results point to a role for RA in promoting GC proliferation at early stages of gonad development. These data are consistent with our hypothesis that the loss of the Crabp2 proteins in zebrafish reduces RA signaling in GCs, resulting in lower levels of GC proliferation and subsequently smaller gonads, thus promoting predominantly male development (Fig. 4S).

**Fig. 5.**
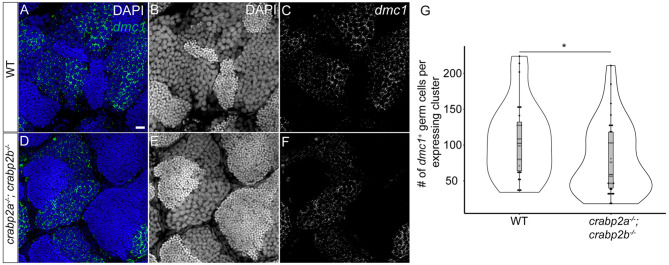
**Retinoic acid function regulates meiotic entry in adult testes.** (A-F) Representative confocal *z*-slices showing *dmc1* mRNA expression in adult testes from wild type (A-C) and *crabp2a^−/−^; crabp2b^−/−^* double mutants (D-F) using isHCR. *dmc1*-expressing germ cells (GCs) are labeled in green (A,D). (C,F) Grayscale; nuclei are stained with DAPI in grey (B,E) and blue (A,D). (G) Violin, and box and whisker plot depicting the number of GCs in a gonad expressing *dmc1*. Each data point represents the total number of GCs in a single *dmc1*-expressing cluster. Four regions were imaged per gonad and gonads from four adults were imaged for wild type and mutants. An unpaired two-sample Wilcoxon test was performed (*P=0.02). The box represents the interquartile range (IQR) where 50% of the data points are present. The height of the box is inversely proportional to the clustering of the measurements. Outliers are present outside the box and quartiles. The horizontal line in the box plot represents the median. Scale bar: 20 μm.

One role for RA in mammalian gonads is in promoting the expression of stimulated by retinoic acid 8 (*Stra8*) in embryonic GCs of the developing ovary. Expression of *Stra8* precedes upregulation of meiotic markers, including *Dmc1* in ovarian GCs (Menke et al., 2003). Recent studies have shown that *dmc1* marks early meiotic stage 1A pre-follicle phase oocytes in developing zebrafish gonads (Liu et al., 2022). To determine whether meiotic entry may be affected in *crabp2a^−/−^; crabp2b^−/−^* double mutants, we assayed the expression of *dmc1* by isHCR in adult testes and found a modest yet significant reduction in its expression, suggesting a potential role of Crabp2a and Crabp2b in regulating meiotic progression (Fig. 5).

In summary, our data reveal crucial roles for Crabp2 proteins and RA in GCs during early gonad development in zebrafish. Our work indicates that Crabp2 proteins specifically promote RA signaling and GC proliferation, which in turn influence the number of oocytes produced during the bipotential stage, which is a crucial factor in maintaining female development and balanced sex ratios. These results are consistent with previous studies, largely *in vitro*, suggesting that Crabp2 localizes to the nucleus and helps to transport RA to its nuclear hormone receptors (RARs and RXRs), in contrast to Crabp1 (Budhu et al., 2001; Ross and Zolfaghari, 2011; Sessler and Noy, 2005). They are consistent with our previous studies showing that Crabp2 proteins, and not Crabp1 proteins, are RA inducible and promote robustness in RA signaling in embryonic zebrafish (Cai et al., 2012). They also provide some of the first evidence for an essential role for members of this extremely evolutionarily conserved family of proteins (Crabp proteins) in development or tissue homeostasis (Gorry et al., 1994; Lampron et al., 1995). While such a role in GC or gonad development has not been demonstrated in other vertebrates, future studies of Crabp-deficient mammals are needed to determine if some aspects are conserved more generally.

Our findings also suggest that RA functions during the period in zebrafish in which GC number shifts from a unimodal distribution at 7 dpf to a bimodal distribution at 14 dpf, and likely also throughout adulthood, when it is required for GC homeostasis (Tzung et al., 2015). Since RA is derived from vitamin A, and food availability is well-known to influence sex ratios in zebrafish (Lawrence et al., 2008), our results are also consistent with an environmental influence. Overall, our work highlights yet another potential gene-environment interaction that contributes to the complex mosaic of GC development and sex determination in zebrafish.

## MATERIALS AND METHODS

### Fish lines

Wild-type AB zebrafish were used in this study. *Tg(RARE-gata2a:NLS-EYFP)^ID1^* transgenics, which we refer to simply as *Tg(RARE:YFP)* in the text, were used to examine RA-responsive cells (Perz-Edwards et al., 2001). Crabp2a antibody staining experiments used transgenic *Tg(piwil1:EGFP)^uc02^* animals (Leu and Draper, 2010). All animals were maintained as described previously (Westerfield, 2000) and in accordance with University of California, Irvine, Institutional Animal Care and Use Committee protocols.

### Generation of Crabp mutants

To generate mutants, we used the CRISPR/Cas9 system, as previously described (Jao et al., 2013). gRNA target sequences were designed using the CHOPCHOPv3 web tool (Labun et al., 2019), targeting exon 2 of *crabp1a* (NCBI Gene ID: 171479), *crabp1b* (NCBI Gene ID: 415102), *crabp2a* (NCBI Gene ID: 171480) and *crabp2b* (NCBI Gene ID: 503502). We used template-based assembly to produce gRNA templates with 5′ primers and universal 3′ primer sequence: AAAAGCACCGACTCGGTGCCACTTTTTCAAGTTGATAACGGACTAGCCTTATTTTAACTTGCTATTTCTAGCTCTAAAAC. For gene-specific 5′ primer sequences and genotyping primers, see Table S1. gRNAs were transcribed using the MEGAshortscript T7 Transcription Kit (Invitrogen, AM1354) and one-cell stage wild-type AB embryos were co-injected with gRNA and Cas9 mRNA (Jao et al., 2013). Mutant alleles produced in this study were *crabp1a* (−10 bp)*^ir1097^*, *crabp1b* (−5 bp)*^ir1098^*, *crabp2a* (−11 bp)*^ir1099^* and *crabp2b* (−65 bp)*^ir1100^*. Mutants were genotyped using a heteroduplex mobility shift assay (Zhu et al., 2014). Briefly, tailfin clips from adult fish were hydrolyzed using 50 mM NaOH at 95°C for 20 min. The pH was adjusted by adding 0.1 volumes of 1 M Tris (pH 8.0). A PCR was performed using genotyping primers (Table S1). The PCR product was analyzed on a 10% native polyacrylamide gel to screen for heteroduplexes. Samples that showed heteroduplex products were further cloned into pGEM-T vectors and the clones sequenced by Sanger sequencing to identify the nature and extent of mutation. Individual mutant alleles were further bred and genotyped using a heteroduplex gel shift assay for every generation.

### Retinoic acid and BrdU treatments

Stocks of 10 mM all-trans RA (Sigma-Aldrich, R2625) were prepared in DMSO and diluted to 0.5 µM RA in embryo medium on the day of treatment. Wild-type or mutant larvae were treated with all-trans RA (Sigma-Aldrich, R2625) or the vehicle DMSO (0.00005%) in embryo medium. Treatments began at 8 dpf in the dark, with daily replacement of treatment medium until fixation with 4% paraformaldehyde (PFA). For BrdU (Sigma-Aldrich, B-5002) treatments, 24 h before fixation, larvae were transferred to a 0.5% DMSO solution of 10 mM BrdU in embryo medium.

### Immunohistochemistry

Larvae were euthanized with tricaine (Sigma-Aldrich, MS-222) and placed on ice. Larvae were decapitated anterior to the pectoral fin and the abdomen was slit using iridectomy scissors along the ventral midline in Ringer's solution. Dissected larvae were fixed with neutral pH-buffered 4% PFA at 4°C overnight. Samples were washed in PBS-DT (0.5% DMSO, 1% Triton x-100) and permeabilized by digestion with proteinase K solution (10 µg/ml at room temperature for 10 min followed by re-fixation with 4% PFA for 20 mins. The larvae were washed again in PBS-DT and blocked with either 5% donkey or goat serum in PBS-DT. Adult fish were similarly euthanized with tricaine in an ice bath. The fish were decapitated anterior to the pectoral fin and the abdomen was slit open along the ventral midline in Ringer's solution. The testes were removed gently using a pair of forceps and fixed immediately in a tube of ice-cold 4% PFA. After overnight fixation, dissected testes were treated similarly to the larvae for antibody staining.

### Antibodies

Primary antibodies in this study were used at the following concentrations: rabbit anti-Ddx4 (formerly called Vasa) 1:2000 (Knaut et al., 2000), mouse anti-BrdU 1:500-1:1000 (Sigma-Aldrich, B-2531), rat anti-pHH3 1:500 (Sigma-Aldrich, H9908), rabbit anti-Crabp2a 1:200 (GeneTex, GTX125986), rabbit anti-actin 1:1000 (GeneTex, GTX637675), donkey anti-rabbit horse radish peroxidase (HRP) (Jackson ImmunoResearch, 711-035-152) and nuclear DAPI staining was performed at a concentration of 1:1000.

### *In situ* hybridization chain reaction

Adult gonads were fixed in 4% PFA overnight at 4°C and dehydrated in 100% methanol at −20°C overnight. An in situ hybridization chain reaction (isHCR) protocol was followed as previously published (Trivedi et al., 2018). The *crabp2a* probe set was custom designed and synthesized by Molecular Instruments and was used at 12 nM concentration to improve the signal. The *dmc1* probe set was a kind gift from the Draper lab (Liu et al., 2022). The isHCR hairpin amplifier used in this assay was B1-Alexafluor 488. After signal development, the gonads were mounted in 50% glycerol/5×SSC and imaged.

### Imaging and germ cell quantification

Larval gonads stained with antibodies were dissected and whole-mounted in 100% glycerol on glass slides with coverslips affixed with vacuum grease for imaging after dehydration in a graded series of progressively higher glycerol concentrations. Confocal imaging was performed on a Leica SP8 confocal with a 40× water immersion objective. At larval stages, all Ddx4-positive cells were counted, regardless of stage. For adult testis quantification, only the highest Ddx4-expressing germ cells, which correspond to spermatogonia based on nuclear morphology, size and Ddx4 staining intensity, were counted. Image processing was performed using ImageJ/Fiji software (Schindelin et al., 2012).

### Quantitative real-time PCR (qRT-PCR)

For each condition, 50 embryos were pooled and homogenized in TRIzol Reagent (Invitrogen, 15596018) using a Beadbug 3 Microtube Homogenizer (Benchmark Scientific, D1030) with 1.0 mm zirconium beads (Benchmark Scientific, D1032-10). RNA was extracted using the standard TRIzol Reagent protocol. Normalized concentrations of RNA were used as input to produce cDNA using the ProtoScript II First Strand cDNA Synthesis Kit (New England BioLabs, E6560L). cDNA was diluted 1:25 in water and used as template in PCR reactions with Luna Universal qPCR Master Mix (New England BioLabs, M3003S). qRT-PCR was performed using a LightCycler 480 Instrument II (Roche) and analyzed with Roche's LightCycler software. Experiments were performed in triplicates from a single biological experiment. Zebrafish Ribosomal Protein S13 (rps13) RNA levels were used for normalization.

### Western blot

Embryos (25 wild type and *crabp2a^−/−^; crabp2b^−/−^*mutants) were euthanized and transferred to ice-cold Radio Immuno-Precipitation Assay (RIPA) buffer [150 mM NaCl, 1% Triton, 0.5% sodium deoxycholate, 50 mM Tris (pH 7.4), 0.1% sodium dodecyl sulphate] containing a protease inhibitor cocktail (Sigma-Aldrich, 11697498001). The larvae were dissociated using a plastic micro pestle. The protein extract was run on a 10% SDS-PAGE gel and assayed for Crabp2a and Actin. The blot was developed using luminol based chemiluminescent assay (Bio-Rad, 1705060S) and imaged using a BioRad ChemiDoc imaging system. The signal density was quantified using the ImageJ gel analyzer tool (Stael et al., 2022).

## Supplementary Material



10.1242/develop.202549_sup1 Supplementary information
