# Supplementary material for: Cellular retinoic acid-binding proteins regulate germ cell proliferation and sex determination in zebrafish
Source: Development. 2024 Dec 13;151(24):dev202549. doi: 10.1242/dev.202549 (PMC11664166; doi:10.1242/dev.202549)
Supplement: Supplementary information [file develop-151-202549-s1.pdf]

**Fig. S1.**

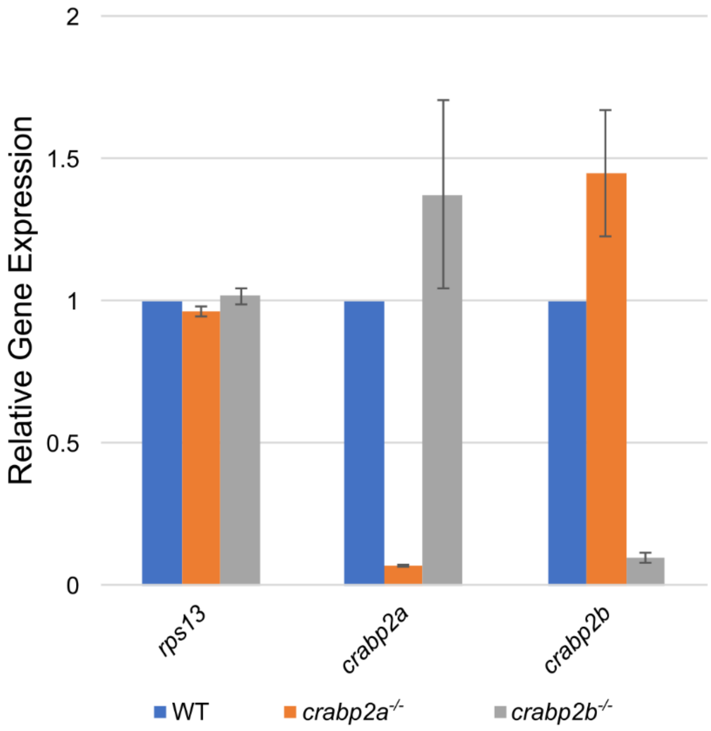


**Fig. S1.** Upregulation of *crabp2* paralogues in *crabp2*$^{-/-}$ mutants. qRT-PCR analysis shows relative fold-change in gene expression of *rps13*, *crabp2a*, and *crabp2b*, in wild-type (WT), *crabp2a*$^{-/-}$, and *crabp2b*$^{-/-}$ embryos at 24 hpf. Several embryos are pooled for each sample.

Development • Supplementary information

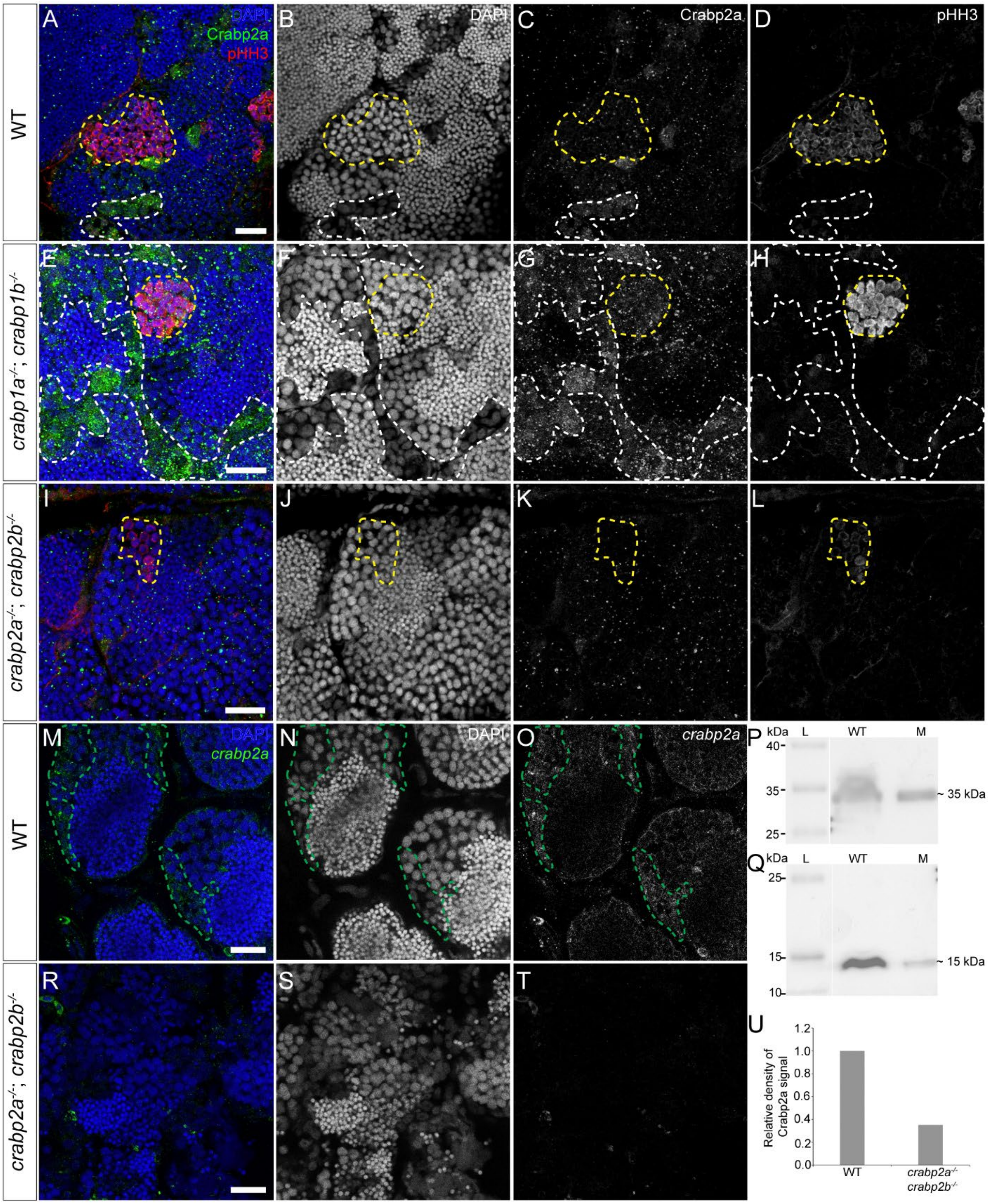


Development • Supplementary information

**Fig. S2. Crabp2a is expressed in germ cells (GCs) of the adult testis.**
(A-L) Representative confocal z-slices of adult WT (A-D), *crabp1a*$^{-/-}$; *crabp1b*$^{-/-}$ double mutant (E-H), *crabp2a*$^{-/-}$; *crabp2b*$^{-/-}$ double mutant (I-L) testes stained with antibodies against Crabp2a (grayscale and green) and phospho-histone H3 (pHH3) (grayscale and magenta) and DAPI (grayscale and blue) to visualize the nuclei. (M-O, R-T) Representative confocal z-slices of adult WT (M-O) and *crabp2a*$^{-/-}$; *crabp2b*$^{-/-}$ double mutant (R-T) testes showing expression of *crabp2a* using isHCR in grayscale and green and DAPI labeling the nuclei in grayscale and blue. (P,Q,U) Western blot on protein extract from WT and *crabp2a*$^{-/-}$; *crabp2b*$^{-/-}$ double mutant (M) larvae using anti-beta Actin antibody (P) and anti-Crabp2a (Q). The ladder has been cropped from the original image and placed adjacent to the appropriate Actin (P) and Crabp2a (Q) blots to serve as a reference for the respective bands. (U) Histogram showing relative density of Crabp2a signal from western blot. The white dashed lines mark ROIs showing GC clusters with high levels of anti-Crabp2a signal. The yellow dashed lines mark ROIs of GC clusters with strong anti-pHH3 signal. The green dashed lines mark ROIs of GC clusters showing expression of *crabp2a* mRNA detected using isHCR. For antibody staining WT – 7 dpf n = 3 larvae, 12 dpf n = 7 fish; *crabp2* mutants – 7 dpf n = 3 larvae, 12 dpf n = 8 fish.  Scale bars = 20 μm for each panel.

Development • Supplementary information

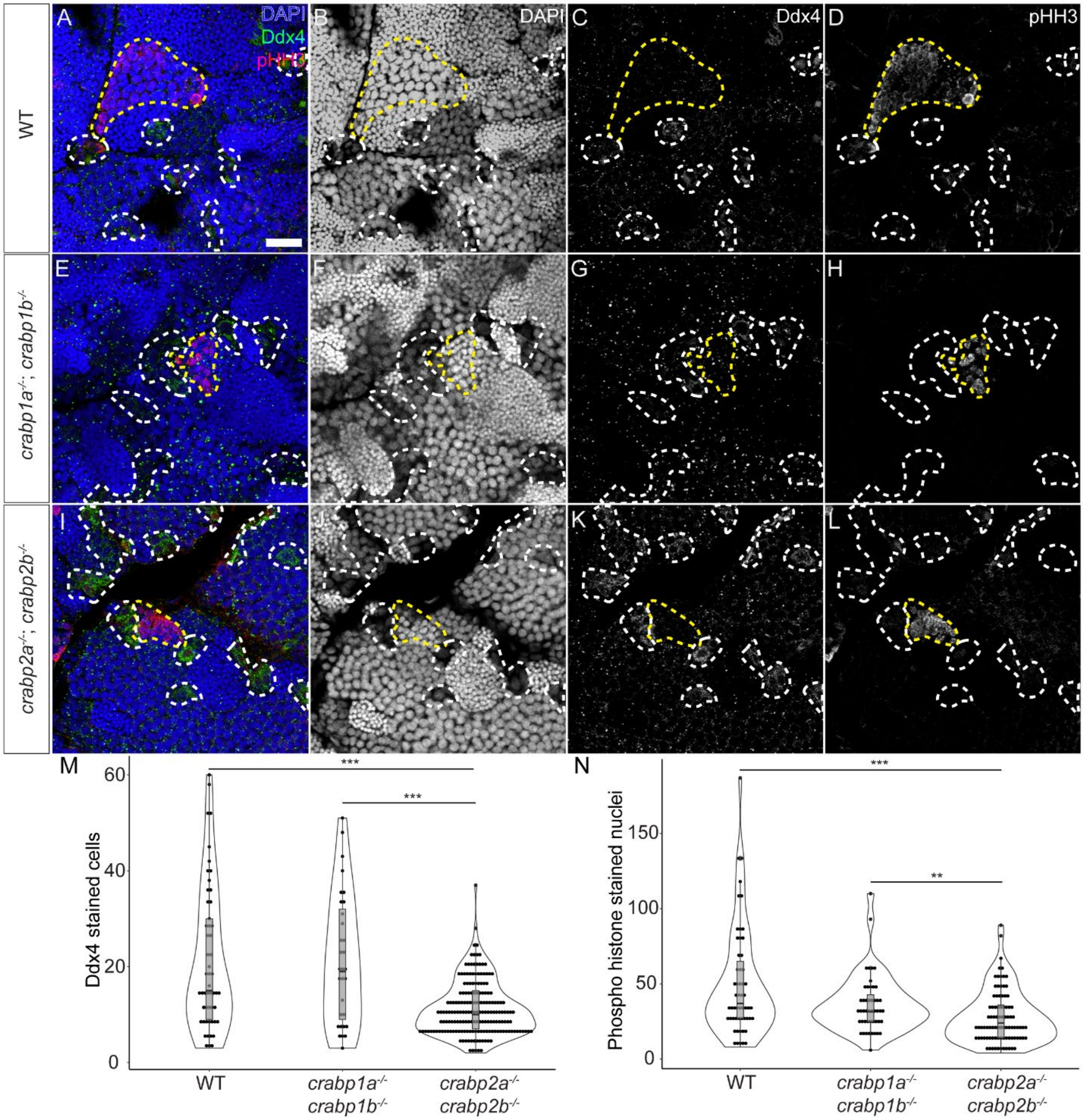


**Fig.S3. Adult Crabp2 mutant testes have reduced GC proliferation and GC number.** (A-L) Representative images of confocal z-slices of anti-Ddx4 and anti-pHH3 antibody staining to show mitotic GCs in adult testes of WT (A-D), *crabp1a*$^{-/-}$*; crabp1b*$^{-/-}$ double mutants (E-H), and *crabp2a*$^{-/-}$*; crabp2b*$^{-/-}$ double mutants (I-L). GCs are labeled with anti-Ddx4 antibody in green and grayscale, proliferating cell nuclei with anti-pHH3 antibody in red and grayscale,

Development • Supplementary information

and nuclei with DAPI in blue and grayscale. (M) Violin and box plots depicting the number of GCs in a gonad by genotype. The box represents the interquartile range (IQR) where 50% of the data points are present. The height of the box is inversely proportional to the clustering of the measurements. Outliers are present outside the box and quartiles. The horizontal line in the box plot represents the median. Each data point represents the total number of anti-Ddx4 stained GCs in one cluster per confocal data set. Each gonad was imaged in 4 – 6 regions and gonads from about 4 adults were imaged in this experiment. *crabp1a*$^{-/-}$*; crabp1b*$^{-/-}$ double mutants and WT animals have similar proportions of Ddx4(+) GCs. *crabp1a*$^{-/-}$*; crabp1b*$^{-/-}$ double mutants have a significantly higher proportion of Ddx4(+) GCs compared to *crabp2a*$^{-/-}$*; crabp2b*$^{-/-}$ MZ double mutants (P = 9.6 *$10^{-05}$). WT animals have a significantly higher number of Ddx4(+) GCs compared to *crabp2a*$^{-/-}$*; crabp2b*$^{-/-}$ MZ double mutants (P = 5.5*$10^{-06}$). (Unpaired two-tailed t-test; ** = P < 0.001, ***= P<0.0001; n.s. = no significance). (N) Violin and box plots depicting the number of anti-pHH3 stained GCs in a gonad by genotype. Each data point represents the number of pHH3(+) GCs in one cluster per confocal data set. WT animals have a significantly higher number of mitotic GCs compared to *crabp2a*$^{-/-}$*; crabp2b*$^{-/-}$ MZ double mutants (P = 2.09*$10^{-05}$). While there is no significant difference between WT and *crabp1a*$^{-/-}$*; crabp1b*$^{-/-}$ double mutants., *crabp1a*$^{-/-}$*; crabp1b*$^{-/-}$ double mutants have higher number of pHH3 stained dividing GCs compared to *crabp2a*$^{-/-}$*; crabp2b*$^{-/-}$ MZ double mutants (P = 0.0028). Each gonad was imaged in 4 – 6 regions and gonads from about 4 adults were imaged in this experiment. Scale bars = 20 μm for each panel.

Development • Supplementary information

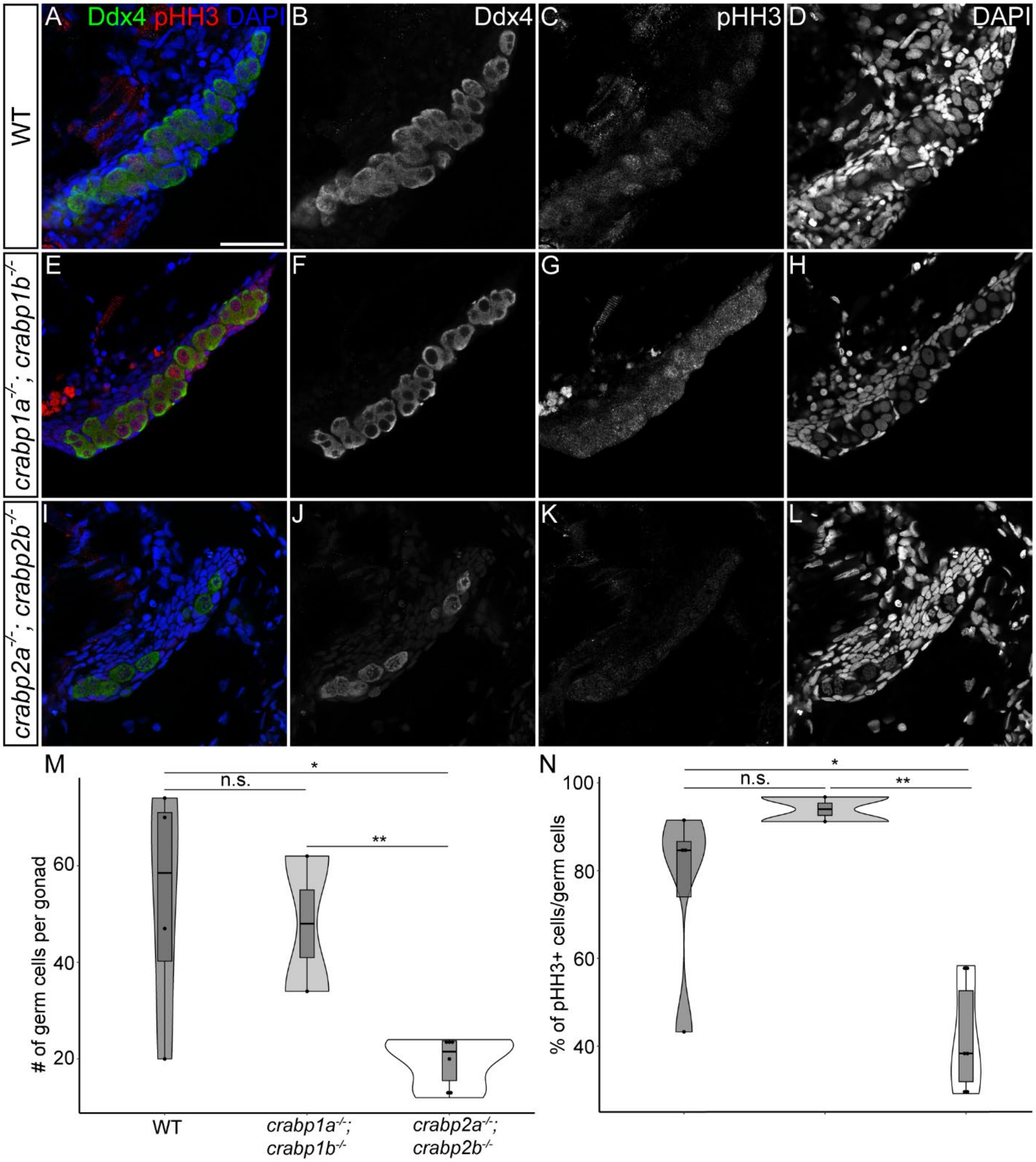


**Fig. S4. Crabp2 mutant gonads have fewer germ cells and decreased proliferation.**

(A-L) Representative confocal images of anti-pHH3 stained 11 dpf gonads of WT (A-D), *crabp1a*$^{-/-}$; *crabp1b*$^{-/-}$ double mutants (E-H), and *crabp2a*$^{-/-}$; *crabp2b*$^{-/-}$ double mutants (I-L). GCs are labeled with anti-Ddx4 antibody in green, proliferating cell nuclei with anti-pHH3 antibody in

Development • Supplementary information

red, and nuclei with DAPI in blue (A, E, I). Grayscale Ddx4 (B, F, J), pHH3 (C, G, K) and DAPI (D, H, L). (M) Violin and box plots depicting the number of GCs in a gonad by genotype. Each data point represents the total number of GCs in one gonad. *crabp1a*$^{-/-}$*; crabp1b*$^{-/-}$ double mutants and WT animals have similar GC numbers (mean ± SEM = 48.00 ± 14, 52.75 ± 12.43, respectively; P = 0.8283). *crabp1a*$^{-/-}$*; crabp1b*$^{-/-}$ double mutants have a significantly higher number of GCs compared to *crabp2a*$^{-/-}$*; crabp2b*$^{-/-}$ MZ double mutants (mean ± SEM = 19.50 ± 2.16; P = 0.01). WT animals have a significantly higher number of GCs compared to *crabp2a*$^{-/-}$*; crabp2b*$^{-/-}$ MZ double mutants (P = 0.0115). (N) Violin and box plots depicting the percentage of proliferating GCs in a gonad by genotype. The box represents the interquartile range (IQR) where 50% of the data points are present. The height of the box is inversely proportional to the clustering of the measurements. Outliers are present outside the box and quartiles. The horizontal line in the box plot represents the median. Each data point represents the number of pHH3(+) GCs in one gonad. *crabp1a*$^{-/-}$*; crabp1b*$^{-/-}$ double mutants and WT animals have similar proportions of pHH3(+) GCs. (mean = 93.96% and 76.00%, respectively; P = 0.3411). *crabp1a*$^{-/-}$ *; crabp1b*$^{-/-}$ double mutants have a significantly higher proportion of pHH3(+) GCs compared to *crabp2a*$^{-/-}$*; crabp2b*$^{-/-}$ MZ double mutants (mean = 41.88%; P = 0.0017). WT animals have a significantly higher number of pHH3(+) GCs compared to *crabp2a*$^{-/-}$*; crabp2b*$^{-/-}$ MZ double mutants (P = 0.0142). (Unpaired two-tailed t-test; * = P < .05, ** = P < .01; n.s. = no significance). Scale bars = 50 µm for each panel. WT – 11 dpf n = 3 larvae; *crabp1* mutants – 11 dpf n = 1 larvae; *crabp2* mutants 11 dpf n = 4 larvae.

Development • Supplementary information

## Table S1. Genotyping primers

| | |
|---|---|
| crabp1a_gRNA | GCAGCTAATACGACTCACTATAGGCGATGCTCCGGAAAGTAGGTTTTAGAGCTAGAA |
| crabp1b_gRNA | GCAGCTAATACGACTCACTATAGCTGAGAAAAGTGGCTTGTGGTTTTAGAGCTAGAA |
| crabp2a_gRNA | GCAGCTAATACGACTCACTATAGGTGATGCTCCGTAAGATTGGTTTTAGAGCTAGAA |
| crabp2b_gRNA | GCAGCTAATACGACTCACTATAGGTGGTCCGAACGCTGGTGGGTTTTAGAGCTAGAA |
| crabp1a_geno_f | TGGGGCAGTTTCAAAGAGTTAT |
| crabp1a_geno_r | CAGTGGTGGAGGTCTTGATGTA |
| crabp1b_geno_f | CAGCTCAGTCAAGAGTGACGAC |
| crabp1b_geno_r | TTGATATAGAACTGCTCCCCGT |
| crabp2a_geno_f | ACATCACTCTCATTCCCCCGA |
| crabp2a_geno_r | TGGGTGTGTGTATACCGTGC |
| crabp2b_geno_f | GCAGACTGGACACGCTCATTA |
| crabp2b_geno_r | GAGAGAATTTGTGGCGTACTGTG |
| crabp2a_qPCR_E3-4_F | GCGAGGGTCCAAAAACCTCA |
| crabp2a_qPCR_E3-4_R | CGTCAGCAGTCATGGTCAGAAT |
| crabp2b_qPCR_E1-2_F | CGGAGAAAACATTCGCGGAC |
| crabp2b_qPCR_E1-2_R | GCTGCTGCAACTGCAATCTT |
| rps13_F | AAACTGGCCAAGAAGGGTCTG |
| rps13_R | GGATTTTGTTGCCAGTGACGA |

gRNA target site sequences are underlined.

Development • Supplementary information
