# Supplementary material for: Cellular retinoic acid-binding proteins regulate germ cell proliferation and sex determination in zebrafish
Source: Development. 2024 Dec 13;151(24):dev202549. doi: 10.1242/dev.202549 (PMC11664166; doi:10.1242/dev.202549)
Supplement: Supplementary file 1 [file develop-151-202549_review_history.pdf]

Cellular retinoic acid binding proteins regulate germ cell proliferation and sex determination in zebrafish

Lianna Fung, Daniel B. Dranow, Arul Subramanian, Natalia Libby and Thomas F. Schilling
DOI: 10.1242/dev.202549

Editor: Steve Wilson

Review timeline

Original submission:	18 November 2023
Editorial decision:	27 December 2023
First revision received:	16 August 2024
Editorial decision:	25 September 2024
Second revision received:	16 October 2024
Editorial decision:	25 October 2024
Third revision received:	29 October 2024
Accepted:	30 October 2024

Original submission

First decision letter

MS ID#: DEVELOP/2023/202549

MS TITLE: Cellular retinoic acid binding proteins regulate germ cell proliferation and sex determination in zebrafish

AUTHORS: Lianna Fung, Daniel B Dranow, and Thomas F Schilling

I have now received all the referees' reports on the above manuscript, and have reached a decision. The referees' comments are appended below, or you can access them online: please go to BenchPressand click on the 'Manuscripts with Decisions' queue in the Author Area.

As you will see, the referees express considerable interest in your work, but have some suggestions for improvements. If you are able to revise the manuscript along the lines suggested, I will be happy receive a revised version of the manuscript. Please also note that Development will normally permit only one round of major revision. If it would be helpful, you are welcome to contact us to discuss your revision in greater detail. Please send us a point-by-point response indicating your plans for addressing the referees' comments, and we will look over this and provide further guidance.

Please attend to all of the reviewers' comments and ensure that you clearly highlight all changes made in the revised manuscript. Please avoid using 'Tracked changes' in Word files as these are lost in PDF conversion. I should be grateful if you would also provide a point-by-point response detailing how you have dealt with the points raised by the reviewers in the 'Response to Reviewers' box. If you do not agree with any of their criticisms or suggestions please explain clearly why this is so.

Reviewer 1

Advance summary and potential significance to field

© 2024. Published by The Company of Biologists under the terms of the Creative Commons Attribution License (https://creativecommons.org/licenses/by/4.0/).

Retinoic acid (RA) is an established regulator of meiotic entry and sex determination in several vertebrates. In mice, RA signaling through Stra8 promotes early meiosis in XX individuals while in XY individuals RA is degraded. In zebrafish the mechanisms regulating sex determination are not fully understood but are known to be influenced by germ cell numbers in the embryo and timing of meiotic entry, with higher numbers of germ cells and early meiotic entry correlating with ovary development and fewer germ cells and later meiotic entry correlating with testis development. However, zebrafish lack Stra8 so the mechanisms regulating germ cell numbers are not clear, but if RA is involved must be mediated by a different factor. In this work, Fung and colleagues provide evidence that zebrafish germ cells express a cellular RA binding protein (Crabp2a) and demonstrate using RARE reporter lines and RA treatment coupled with BRDU assays to show that germ cells are retinoic acid responsive and proliferate in response to RA.
In addition, they generated mutants disrupting four zebrafish cellular RA binding proteins (Crabps: Crab1a,1b and Crab2a, 2b), which solubilize RA and transport it to promote signaling via its nuclear receptors or to degrade it via cytoplasmic degradation enzymes. The data provided indicate the duplicated Crabs, Crab1a and 1b and Crab2a and 2b are redundant to one another and demonstrate that Crab2s but not Crab1s are required for germ cell proliferation upstream of sex determination. Specifically, loss of Crab2s leads to diminished germ cell numbers due to reduced proliferation based on BRDU and PHH3 assays, smaller gonads, and consequently all double mutants develop testis. This study uncovers a previously unappreciated role for RA and Crab2s in regulating germ cell numbers in zebrafish and raise the question of whether this role for Crabps might be conserved. The paper is well written, the images are beautiful, and the data are clearly presented overall. The findings will be of interest to developmental biologists, reproductive biologists and those interested in RA signaling more generally. The main concern is that throughout the paper the numbers of individuals examined or pooled are not clearly stated and there are some details missing from the methods.
These points are detailed below.

Comments for the author

Major:
1) While numbers and percentages are clearly stated for the sex ratio graphs, numbers are missing elsewhere. Specifically, for the image data in Figs 1C-H, Fig. 2, Fig. 3, Fig. 4. In Figs. 3Q,R, 4Q,R, S2 M,N box and whisker plots are shown; however, violin or pirate plots would provide better visualization of the density spread/distribution, and outliers.
2) Is the Crab2a antibody specific for Crab2a or does it also recognize Crab2b? Were single mutants examined to validate this?
3) As the gonad develops the germ cells enter different stages and begin to differentiate and form germline cysts. Based on the Ddx4 shown in the various experiments, the germ cells appear to be in different stages (some have punctate Ddx4 and others more diffuse and some appear to be in cysts while others do not).
Please indicate in the methods section how GC numbers were quantified. Were all cells expressing Ddx4 counted as germ cells regardless of stage (e.g., cyst or non-cyst)?
4) Please indicate how many embryos per genotype were pooled for the qRT experiments.
5) Is all signaling in the germline through Crab2? More specifically, does RA treatment have any effect on Crab2 double mutants?

Minor but important:
1) Did 2a mutants that were het for 2b or vice versa show any indication of a dosage effect or are the genes fully redundant?
2) Although primers are provided, the methods section does not indicate how mutants were genotyped for the various assays. Were they sequenced or detected based on size difference on a gel? Please indicate this in the methods section.
3) In the methods section, the %DMSO should be stated for the RA experiments as it was for the BRDU experiments.

<u>Reviewer 2</u>

Advance summary and potential significance to field

© 2024. Published by The Company of Biologists under the terms of the Creative Commons Attribution License (https://creativecommons.org/licenses/by/4.0/).

In this manuscript, Fung et al. detail the phenotype of loss-of-fucntion mutations in the cellular retinoic acid (RA) binding proteins (Crabps), crabp2a and crabs2b, in zebrafish. Crabps proteins bind RA and aid in both its transport the RA receptors, or the RA degradation enzymes. RA signaling is known to play many important roles in patterning of the early vertebrate embryo, and has also been found to positively regulate germ cells entrance into meiosis. They find that single mutants in either crabp2a or crabp2b have no phenotype, but double mutants develop as all male, suggesting a role in gonad development or function.
Further analysis shows that the gonads of double mutants are smaller than wildtype gonads as early as 12 days postfertilization (dpf) and also contain fewer germ cells. Using transgenic RA-responsive reporter lines they show evidence that early germ cells are RA responsive, and in the absence of crabp2a/b have reduced proliferation relative to wildtype germ cells arguing that RA-signaling is necessary for normal germ cell proliferation. Finally, the show that treating 10-12 dpf larval fish with exogenous RA increases their proliferation, arguing that RA is sufficient to promote early germ cell proliferation. They conclude that RA signaling plays a role in regulating early germ cell proliferation and thus appears to play a key role in influencing sex determination in zebrafish.

The data presented support the major conclusions of the paper. They also generally fit with what has already been reported regarding the expression of the genes encoding RA metabolic enzymes and receptors in the zebrafish gonad.
Importantly, they provide the first hint that RA signaling in zebrafish may be playing a similar role during germ cell development to what has been established in mammals. However, because the manuscript does not present much beyond a cursory examination of the phenotype (germ cell proliferation and sex ratios), it is unclear if the role of RA in zebrafish germ cell development is analogous or different to that in mammals.

Comments for the author

Major comments/suggestions:
1) crabp2a and crabp2b are expressed in many cell types during early development and likely is many tissues in larval fish. However, the expression of either gene in the gonad was not clearly established. The authors used an anti-Crabp2a antibody that showed apparent weak staining in wild-type germ cells, but they did not establish the specificity of this antibody by staining crabp2a (or crabp2b) mutants. Published scRNA-seq data shows expression of crabp2b and crabp2b in premeiotic germ cells and a subset of stromal cells in the 40 dpf ovary. It would therefore be nice to see higher resolution immunofluorescence to see if other cell types in addition to germ cells express Crabp2a.
2) The present study shows double mutant germ cells proliferate less than wild-type germ cells. In mice, RA signaling promotes entry into meiosis. There are now several established marker genes for zebrafish cells that have entered meiosis that could be used to compare the relative number of these cells between mutant, wild-type, and RA treated gonads (e.g. dmc1, sycp3). This analysis would allow for a comparison to the role of RA in the mammalian gonad.
3) A Tg(RARE:nls-YFP) transgenic line was used to establish that wild-type germ cells are RA responsive. However, it appears that nls-YFP localizes to the cytoplasm, and not the nucleus (Fig. 2). How do the authors interpret this?

Minor comments/suggestions (in relative order of appearance in manuscript):

1. “…(RA) is a cell-cell signaling molecule that plays an important role in sex determination…” Should be “…sex differentiation…” as this reviewer is not aware of any data showing a role of RA in sex determination.
2. It is not very clear from the text what genetics leads to the increased male phenotype. Is it Zcrabp2a;crabp2b and/or MZcrabp2a;crabp2b mutants that are mostly male?
3. Fig 1: What are the N’s for each of the phenotypic classes analyzed in C-H?
4. “These results are consistent with previous studies, largely in vitro suggesting that Crabp2 localizes to the nucleus and helps transport RA to its nuclear hormone receptors (RARs and RXRs),…” The Crabp2a antibody staining appears to show germ cells localization, but Crabp2a appears to localize to the cytoplasm. This again begs for the control experiment to confirm this antibody recognizes Crabp2a. Does zebrafish Crabp2a have an NLS?
5. Can exogenous RA rescue the double mutant phenotype (i.e. restore germ cell proliferation), or is the effect of exogenous RA also dependent on the function of Crabp2a/b?

© 2024. Published by The Company of Biologists under the terms of the Creative Commons Attribution License (https://creativecommons.org/licenses/by/4.0/).

Reviewer 3

Advance summary and potential significance to field

The authors report an interesting new finding that disruption of the retinoic acid (RA) signaling pathway via mutation of the proteins that deliver RA to its nuclear receptors (crabp2a-/-; crabp2b-/-) leads to a male bias in the sex ratio of zebrafish. It is established that lower numbers of germ cells lead to male development in zebrafish, whereas high numbers of germ cells promote the female fate. The authors present convincing evidence that there are fewer germ cells in crabp2a-/-; crabp2b-/- mutants. They argue that this is the result of a direct effect of RA on germ cell proliferation.

Comments for the author

The argument that RA acts directly on germ cells in this model relies on detection of both the RA reporter and Crabp2a protein in germ cells (Fig. 2). Neither of these images is compelling. The BrdU labeling in Fig. 3 does not appear to be in the large round nuclei of Ddx4+ cells, which is strange. Maybe a blow-up would clarify this point. Even after treatment with RA in Fig. 4, it is still difficult to see nuclear localization of the BrdU signal.

Are the authors convinced that this is a direct effect on GCs? The authors suggest the increase in proliferation occurs within the 7-12 dpf window. It would be useful to present a timecourse to see exactly when RA acts.

There is a section of the text that needs clarification:
"We noticed that both DMSO- and RA-treated larvae had reduced numbers of GCs and BrdU incorporation at 12 dpf compared to untreated fish (compare Figs. 3Q-R, 4Q-R). This may have been due to larvae being kept in the dark to prevent RA degradation and a potential reduction in food intake, as the medium was changed daily several hours after their daily feeding with live rotifers. Despite this, we did not detect significant differences in GC number or proliferation between either condition at 10 dpf."
I did not understand why keeping the fish in the dark or changing the medium affected the outcome, or why "Despite this, we did not detect significant differences". What does this mean?
The point that RA is an "environmental" dietary metabolite that contributes to sex determination in zebrafish is very exciting. While I think the overall finding that loss of crabpb2a and crabp2b affect GC number is solid, it would be valuable for the field to confirm whether or not this is a direct effect, and to determine when in development it occurs.

First revision

Author response to reviewers' comments

Point-by-point responses to reviewers' comments:
Reviewer 1 Comments for the Author:
Major:
1) While numbers and percentages are clearly stated for the sex ratio graphs, numbers are missing elsewhere. Specifically, for the image data in Figs 1C-H, Fig. 2, Fig. 3, Fig. 4. In Figs. 3Q,R, 4Q,R, S2 M,N box and whisker plots are shown; however, violin or pirate plots would provide better visualization of the density, spread/distribution, and outliers.
We agree and now include sample numbers for each experiment in the figure legends. We have replaced the quantification with violin plots including all data points as well as box plots to highlight significant quartiles in the data.
2) Is the Crab2a antibody specific for Crab2a or does it also recognize Crab2b? Were single mutants examined to validate this?

© 2024. Published by The Company of Biologists under the terms of the Creative Commons Attribution License (https://creativecommons.org/licenses/by/4.0/).

We thank the reviewer for raising this important point. The antibody used to detect Crabp2a in this study is a commercial antibody that was raised against a proprietary epitope region. The manufacturer provides an image of an early stage zebrafish embryo with signal detected in the hindbrain retina, spinal cord, and posterior pharyngeal arches in a pattern identical to previously published crabp2a in situ hybridization patterns (see https://zfin.org/ZDB-IMAGE-021105-37#image for an example). In contrast, at approximately the same stage, Crabp2b has specific expression in the anterior-most pharyngeal arches and otic vesicles (see https://zfin.org/ZDB-IMAGE-050208-286#image for an example). These observations are consistent with the antibody recognizing Crabp2a but not Crabp2b.

We have independently tested this antibody in whole gonads and western blots. In addition, we have also analyzed sequence similarities between Crabp1a, Crabp1b, Crabp2a and Crabp2b proteins - they collectively share >60% identity across the entire protein and between Crabp2a and Crabp2b sequences the identity is >70%. Our western blot shows that there is a weak signal in *crabp2a/b* double mutants, corresponding to the WT Crabp2a band. This suggests that there is a weak interaction with the Crabp1 proteins. Since antibody interaction with the Crabp1 proteins is weak (3-fold less in mutants than in WT), it does help us to view the level of Crabp2 proteins in *crabp2* double mutants. While we did not specifically stain single mutants, from our in silico and western analyses, we expect that the antibody most likely would interact with the complementary *crabp2* gene in the single mutant lines. We have added this information results section (lines : 226-233)

3) As the gonad develops the germ cells enter different stages and begin to differentiate and form germline cysts. Based on the Ddx4 shown in the various experiments, the germ cells appear to be in different stages (some have punctate Ddx4 and others more diffuse and some appear to be in cysts while others do not). Please indicate in the methods section how GC numbers were quantified. Were all cells expressing Ddx4 counted as germ cells regardless of stage (e.g., cyst or non-cyst)?

In the larval stages since the gonads are small in size, all Ddx4-positive germ cells were counted, regardless of stage. For adult testis quantification, only the highest Ddx4-expressing germ cells, which correspond to spermatogonia based on nuclear morphology, size, and Ddx4 staining intensity, were counted. Clusters where Ddx4 signal was very weak or appeared punctate were not counted.

4)Please indicate how many embryos per genotype were pooled for the qRT experiments.

The qPCR experiments were performed in triplicates from a single biological experiment. 50 embryos were used for each condition to extract the RNA for the experiment.

5)Is all signaling in the germline through Crab2? More specifically, does RA treatment have any effect on Crab2 double mutants?

We thank the reviewer for raising this issue. We have shown in previous studies that exogenous RA treatment can rescue hindbrain defects in crabp2a and crabp2b antisense morpholino-injected embryos (Cai et al., 2012) and we expect a similar rescue with RA treatment of mutant larvae. We have tried several times but have been unsuccessful performing this experiment, obtaining only negative results. This is likely due to logistical issues in raising and RA treatment of the larvae as well as pinpointing the precise time window necessary for hitting the unimodal to bimodal shift in germ cells for the treatment to cause the change in sex ratio.

Minor but important:

1) Did 2a mutants that were het for 2b or vice versa show any indication of a dosage effect or are the genes fully redundant?

Unfortunately, we did not closely examine crabp2a-/-; crabp2b+/- or crabp2a+/-; crabp2b-/- animals but we did not notice any obvious phenotype with regards to sex ratios. From the qPCR analysis, it appears that either crabp2a or crabp2b is upregulated when its other paralogue is knocked out. We could interpret this as a dosage compensation for the loss of either gene. However, we have not assayed the expression levels of either crabp2a or crabp2b in the heterozygotes as the isHCR signals for these genes are both very weak even when the isHCR probe concentration is increased by 3-fold, making it extremely difficult to perform digital HCR and quantify the expression changes.

2) Although primers are provided, the methods section does not indicate how mutants were genotyped for the various assays. Were they sequenced or detected based on size difference on a gel? Please indicate this in the methods section.

We thank the reviewer for highlighting this missing information, we have now included the

© 2024. Published by The Company of Biologists under the terms of the Creative Commons Attribution License (https://creativecommons.org/licenses/by/4.0/).

information on genotyping in the methods section, lines 105-113 and have added animal numbers to figure legends.
3) In the methods section, the %DMSO should be stated for the RA experiments as it was for the BRDU experiments.
We thank the reviewer for highlighting this missing information, we have now included this information in the methods section. We prepared 10mM RA stocks in pure DMSO then diluted the RA in fish water/medium for experiments so the percentage of DMSO in RA experiments is much lower at 0.00005% for our 0.5uM treatment conditions. We have added this information in the methods section (lines: 116-121).

Reviewer 2 Comments for the Author:
Major comments/suggestions:
1) crabp2a and crabp2b are expressed in many cell types during early development and likely is many tissues in larval fish. However, the expression of either gene in the gonad was not clearly established. The authors used an anti-Crabp2a antibody that showed apparent weak staining in wild-type germ cells, but they did not establish the specificity of this antibody by staining crabp2a (or crabp2b) mutants. Published scRNA-seq data shows expression of crabp2b and crabp2b in premeiotic germ cells and a subset of stromal cells in the 40 dpf ovary. It would therefore be nice to see higher resolution immunofluorescence to see if other cell types in addition to germ cells express Crabp2a.
The commercial crabp2a antibody and crabp2a isHCR probe set both show very weak signals in whole-mounted larvae. Hence, we used adult gonads for the assays and show Crabp2a localization in multiple germ cell populations. Expression is highest in germ cells that have not differentiated into mature sperm. We have included the data as Fig S2 and describe it in the Results section (lines: 239-243)
2)The present study shows double mutant germ cells proliferate less than wild-type germ cells. In mice, RA signaling promotes entry into meiosis. There are now several established marker genes for zebrafish cells that have entered meiosis that could be used to compare the relative number of these cells between mutant, wild-type, and RA treated gonads (e.g. dmc1, sycp3). This analysis would allow for a comparison to the role of RA in the mammalian gonad.
We thank the reviewer for this excellent suggestion. We have obtained the dmc1 probe set as a gift from the Draper lab and performed isHCR on adult gonads. We do see a modest yet significant reduction in dmc1 expressing cells in the crabp2a/b mutants compared to WT adults suggesting reduced GC differentiation potential. This has uncovered additional effects of RA signaling and Crabp2 on GC differentiation in zebrafish, allowing better comparisons with its roles in mammals, and has greatly improved our paper, We have added these data as Fig S5 and included the explanation in the manuscript (lines : 297-304)
3)A Tg(RARE:nls-YFP) transgenic line was used to establish that wild-type germ cells are RA responsive. However, it appears that nls-YFP localizes to the cytoplasm, and not the nucleus (Fig. 2). How do the authors interpret this?
Our previous studies using the RARE:nls-YFP transgene have consistently shown some cytoplasmic localization in addition to cell nuclei, for example in cells of the embryonic hindbrain and spinal cord (White et al., 2007). Nuclear versus cytoplasmic YFP fluorescence may be more difficult to distinguish in some cell types as opposed to others depending for example on their size, and we interpret this as suggesting that there is some inefficiency in the ability of the NLS to target the transgenic protein to nuclei or the ability of NLS to target the nucleus is affected by YFP folding.
.
Minor comments/suggestions (in relative order of appearance in manuscript):
1. (RA) is a cell-cell signaling molecule that plays an important role in sex determination…" Should be "…sex differentiation…" as this reviewer is not aware of any data showing a role of RA in sex determination.
We have changed the word from "determination" to "differentiation" (line 40).
2. It is not very clear from the text what genetics leads to the increased male phenotype. Is it Zcrabp2a;crabp2b and/or MZcrabp2a;crabp2b mutants that are mostly male?
We noticed that in zygotic crabp2a/b double mutants, there were higher numbers of males, but this imbalance was significantly higher in the MZ mutants. Hence, we focused on the MZ mutants for further studies of the functional roles of Crabp2a and 2b.
3. Fig 1: What are the N's for each of the phenotypic classes analyzed in C-H?

© 2024. Published by The Company of Biologists under the terms of the Creative Commons Attribution License (https://creativecommons.org/licenses/by/4.0/).

We have updated the N numbers for each experiment in the figure legends.
4. "These results are consistent with previous studies, largely in vitro, suggesting that Crabp2 localizes to the nucleus and helps transport RA to its nuclear hormone receptors (RARs and RXRs),…" The Crabp2a antibody staining appears to show germ cells localization, but Crabp2a appears to localize to the cytoplasm. This again begs for the control experiment to confirm this antibody recognizes Crabp2a. Does zebrafish Crabp2a have an NLS?
We agree that the Crabp protein localization is confusing. Crabps belong to a larger family of intracellular lipid-binding proteins. Crabps do not have a traditional NLS motif and are present both in the cytosol and nucleus. Cytosolic Crabps (Apo- Crabp) bind with RA in the cytosol that causes a conformational change leading to formation of a domain of basic residues on the surface (Holo-Crabp), which resembles a classical NLS motif in 3D conformation. This domain interacts with importin leading to nuclear localization (Sessler and Noy 2205). Our antibody staining also shows that Crabp2 localizes in the cytosol and nucleus consistent with localization of the Apo and Holo forms of the protein.
5. Can exogenous RA rescue the double mutant phenotype (i.e. restore germ cell proliferation), or is the effect of exogenous RA also dependent on the function of Crabp2a/b?
Please refer to the response to Reviewer 1 (5)

Reviewer 3 Comments for the Author:
The argument that RA acts directly on germ cells in this model relies on detection of both the RA reporter and Crabp2a protein in germ cells (Fig. 2). Neither of these images is compelling. The BrdU labeling in Fig. 3 does not appear to be in the large round nuclei of Ddx4+ cells, which is strange. Maybe a blow-up would clarify this point. Even after treatment with RA in Fig. 4, it is still difficult to see nuclear localization of the BrdU signal.
We have not been able to stain for Crabp2a protein and Ddx4 in the same sample as both antibodies are raised in Rabbit. We have tried to stain larvae from transgenic lines to view Crabp2a but the as mentioned in response to Reviewer 2 (1), the antibody to Crabp2a does not stain well in larvae. We have now included magnified insets in each figure panel for Fig 3. We have also added arrowhead that mark the germ cell nuclei showing localization of BrDU.
Are the authors convinced that this is a direct effect on GCs? The authors suggest the increase in proliferation occurs within the 7-12 dpf window. It would be useful to present a timecourse to see exactly when RA acts.
We agree that this would be an informative experiment, however we were unfortunately due to difficulty in obtaining female crabp2a/2b double mutant female fish, we were unable to generate the crabp2a/2b double mutant animals required for this.
There is a section of the text that needs clarification:
"We noticed that both DMSO- and RA-treated larvae had reduced numbers of GCs and BrdU incorporation at 12 dpf compared to untreated fish (compare Figs. 3Q-R, 4Q-R). This may have been due to larvae being kept in the dark to prevent RA degradation and a potential reduction in food intake, as the medium was changed daily several hours after their daily feeding with live rotifers. Despite this, we did not detect significant differences in GC number or proliferation between either condition at 10 dpf."
I did not understand why keeping the fish in the dark or changing the medium affected the outcome, or why "Despite this, we did not detect significant differences". What does this mean?
We agree that this explanation of RA treatment conditions was confusing as written. To clarify we now say "In Figs. 3Q-R, larvae were maintained with a normal day/night light cycle and continuously fed live rotifers. However, for the experimental conditions in Figs 4Q-R, to prevent light-induced RA degradation, both RA-treated and DMSO-treated larvae were kept in the dark. Additionally, to try and keep RA levels relatively constant over the course of the treatments, the medium/water the larvae were kept in was replaced daily, reducing the amount of available live food. Both the dark conditions and reduced food access may have indirectly influenced the results."
The point that RA is an "environmental" dietary metabolite that contributes to sex determination in zebrafish is very exciting. While I think the overall finding that loss of crabpb2a and crabp2b affect GC number is solid, it would be valuable for the field to confirm whether or not this is a direct effect, and to determine when in development it occurs.
We agree that it would be interesting to know exactly when during development this occurs. However, this would require removing RA from the diet and re-introducing it at varying time points. We have looked into acquiring a vitamin A-deficient zebrafish diet but have been unable to find a manufacturer that can do this.

© 2024. Published by The Company of Biologists under the terms of the Creative Commons Attribution License (https://creativecommons.org/licenses/by/4.0/).

References
1. **Cai, A.Q., Radtke, K., Linville, A., Lander, A.D., Nie, Q., Schilling, T.F.** (2012). Cellular retinoic acid-binding proteins are essential for hindbrain patterning and signal robustness in zebrafish. *Development* **139**, 2150-2155.
2. **White, R.J., Nie, Q., Lander, A.D., Schilling, T.F.** (2007). Complex regulation of cyp26a1 creates a robust retinoic acid gradient in the zebrafish embryo. *PLoS Biol.* **11**, e304.

Second decision letter

MS ID#: DEVELOP/2023/202549

MS TITLE: Cellular retinoic acid binding proteins regulate germ cell proliferation and sex determination in zebrafish

AUTHORS: Lianna Fung, Daniel B Dranow, Arul Subramanian, Natalia Libby, and Thomas F Schilling

I have now received all the referees reports on the above manuscript, and have reached a decision. The referees' comments are appended below, or you can access them online: please go to BenchPressand click on the 'Manuscripts with Decisions' queue in the Author Area.

The reviewers are mostly happy with your revisions and we would like to publish a revised manuscript in Development, provided that the remaining,relativelyminor comments are addressed. Please attend to all of the reviewers' comments in your revised manuscript and detail them in your point-by-point response. If you do not agree with any of their criticisms or suggestions explain clearly why this is so.

Reviewer 1

Advance summary and potential significance to field

The authors have addressed the concerns raised in the previous submission and have added new data and details to the revised manuscript. While some questions remain open, in part due to technical limitations or because they are beyond the scope of this work such as the question of whether this role for Crabps might be conserved, this study uncovers a previously unappreciated role for RA and Crab2s in regulating germ cell numbers in zebrafish. The paper is well written, the images are beautiful, and the data are clearly presented overall. The findings will be of interest to developmental biologists, reproductive biologists and those interested in RA signaling more generally. I have only a couple of minor points that the authors may wish to address.

Comments for the author

Minor:
1) Page 12 "Notably, laboratory larval zebrafish tend to have a unimodal distribution of GCs in the population at 7 dpf but by 14 dpf a bimodal shift distinguishes larvae with higher GC numbers, which are more likely to develop as females (Tzung et al., 2015)." Consider revising this sentence or adding an additional sentence to define what is meant by unimodal and bimodal in terms of the GC phenotypes as this shift might not be clear to all readers.

2) For supp. Fig 5D, consider changing the axis label to "dmc1+ cells per expressing cluster" or "# cells in dmc1 expressing cluster" to more clearly describe what the graph is showing.

Reviewer 2

© 2024. Published by The Company of Biologists under the terms of the Creative Commons Attribution License (https://creativecommons.org/licenses/by/4.0/).

Advance summary and potential significance to field

In this revised manuscript, Fung et al. detail the phenotype of loss-of-function mutations in the cellular retinoic acid (RA) binding proteins (Crabp), crabp2a and crabs2b, in zebrafish. Crabp proteins bind RA and aid in both its transport the RA receptors, or the RA degradation enzymes. RA signaling is known to play many important roles in patterning of the early vertebrate embryo, and has also been found to positively regulate germ cells entrance into meiosis. They find that single mutants in either crabp2a or crabp2b have no phenotype, but double mutants develop as all male, suggesting a role in gonad development or function.
Further analysis shows that the gonads of double mutants are smaller than wildtype gonads as early as 12 days postfertilization (dpf) and also contain fewer germ cells. Using transgenic RA-responsive reporter lines they show evidence that early germ cells are RA responsive, and in the absence of crabp2a/b have reduced proliferation relative to wildtype germ cells arguing that RA-signaling is necessary for normal germ cell proliferation. Finally, they show that treating 10-12 dpf larval fish with exogenous RA increases their proliferation, arguing that RA is sufficient to promote early germ cell proliferation. They conclude that RA signaling plays a role in regulating early germ cell proliferation and thus appears to play a key role in influencing sex determination in zebrafish.

A role for RA in germ cell biology of mammals is well established, but this is the first direct evidence that RA regulates aspects of germ cell biology in teleost. Although it is still not clear if the roles of RA in mammalian and teleost germ cells are analogous, this work is a good first step towards address this gap in knowledge.

Comments for the author

The revised manuscript does a better job placing this work in the broader context of the field and addressed all the concerns of this reviewer.

Reviewer 3

Advance summary and potential significance to field

It is established that germ cell number influences sex determination in zebrafish. This manuscript presents evidence that retinoic acid (RA) affects sex determination in zebrafish by influencing germ cell proliferation. The strongest evidence comes from fish carrying mutations in the retinoic acid binding proteins, crabp2a and crabp2b. These fish show a strongly male-biased sex ratio, with fewer germ cells, and smaller gonads.

RA is known to mediate entry into meiosis in germ cells in both sexes. However, RA has also been shown to influence somatic sex determination in mammals by several groups (Bowles et al. Cell Rep. 2018; Minkina et al., DevCell 2014). The authors argue that the affect they report is mediated by Crabp2 expression in germ cells rather than in somatic cells in the gonad. However, this conclusion is less convincing based on results presented in the figures. It is important to the field to be confident about this conclusion. Alternatively, the authors might want to leave open the possibility that RA (also) acts by regulating somatic fate in zebrafish gonads.

Comments for the author

Fig. 2 – The authors attempt to show that both RA signaling and Crabp2a are localized to germ cells. Better images are needed here to support this claim. The YFP reporter for RA signaling is very weak at both 12 and 23 dpf. At 13 dpf, the investigators use the antibody against Crabp2a to demonstrate that this gene is expressed in germ cells. This stain is also very weak and difficult to interpret.

Fig. 3 -- Here the authors attempt to show that proliferation is reduced in germ cells using BrdU incorporation. However, I have a difficult time seeing BrdU labeling in germ cells of wild type gonads on 7dpf, whereas I see a major reduction in somatic cell proliferation in crabp2a mutants at

© 2024. Published by The Company of Biologists under the terms of the Creative Commons Attribution License (https://creativecommons.org/licenses/by/4.0/).

this stage. At 12dpf, BrdU incorporation in wild type germ cell nuclei is more convincing, but I don't see a big difference in mutant gonads.

Fig. 4 – Addition of RA to culture medium does increase gonad size and BrdU incorporation, but again this seems to occur most obviously in nuclei surrounding germ cells. Higher magnification images of germ cell compared to somatic cell nuclei might alter this interpretation.

Second revision

Author response to reviewers' comments

Point by point Response to Reviewers : DEVELOP/2023/202549

Reviewer 1

Minor:
1) Page 12 "Notably, laboratory larval zebrafish tend to have a unimodal distribution of GCs in the population at 7 dpf but by 14 dpf a bimodal shift distinguishes larvae with higher GC numbers, which are more likely to develop as females (Tzung et al., 2015)." Consider revising this sentence or adding an additional sentence to define what is meant by unimodal and bimodal in terms of the GC phenotypes as this shift might not be clear to all readers.

We have revised the sentence as follows (lines 248-252): "Notably, laboratory larval zebrafish tend to have a unimodal distribution of GCs in the population at 7 dpf (exhibiting a normal distribution of GCs in a population around a single peak) but by 14 dpf a bimodal shift (exhibiting a distribution of GCs in the population around two peaks) distinguishes larvae with higher GC numbers, which are more likely to develop as females (Tzung et al., 2015)."

2) For supp. Fig 5D, consider changing the axis label to "dmc1+ cells per expressing cluster" or "# cells in
dmc1 expressing cluster" to more clearly describe what the graph is showing.

We thank the reviewer for this suggestion and we have changed the labeling on the figure.

Reviewer 2

The revised manuscript does a better job placing this work in the broader context of the field and addressed all the concerns of this reviewer.

We thank the reviewer for their comment and previous constructive criticisms, which have improved our paper.

Reviewer 3

It is established that germ cell number influences sex determination in zebrafish. This manuscript presents evidence that retinoic acid (RA) affects sex determination in zebrafish by influencing germ cell proliferation. The strongest evidence comes from fish carrying mutations in the retinoic acid binding proteins, crabp2a and crabp2b. These fish show a strongly male-biased sex ratio, with fewer germ cells,and smaller gonads.

1) RA is known to mediate entry into meiosis in germ cells in both sexes. However, RA has also been shown to influence somatic sex determination in mammals by several groups (Bowles et al. Cell Rep. 2018; Minkina et al., Dev Cell 2014). The authors argue that the affect they report is mediated

© 2024. Published by The Company of Biologists under the terms of the Creative Commons Attribution License (https://creativecommons.org/licenses/by/4.0/).

by Crabp2 expression in germ cells rather than in somatic cells in the gonad. However, this conclusion is less convincing based on results presented in the figures. It is important to the field to be confident about this conclusion. Alternatively, the authors might want to leave open the possibility that RA (also) acts by regulating somatic fate in zebrafish gonads.

The reviewer is correct that evidence in mice suggests that sex differentiation requires continuous reinforcement from somatic cells as loss of somatic cell-expressed Dmrt1 results in male to female transdifferentiation of the XY somatic gonad (Matson et al., 2011) and, conversely that loss of Foxl2 results in female-to-male transdifferentiation in the XX somatic gonad (Uhlenhaut et al., 2009). There is evidence that a similar mechanism is conserved in vertebrates, including zebrafish (Dranow et al., 2016; Webster et al., 2017; Romano et al., 2020). Importantly, our data show that crabp2a/b mutants can produce oocytes during the bipotential stage, suggesting that the somatic environment is capable of supporting oocyte development. By contrast, other examples of sex-reversal due to dysfunction of factors expressed in somatic cells result in either gradual sex reversal over time (e.g. female to male) in both mice and fish or the near complete loss of oocytes early during bipotential stages (such as in cyp19a1a mutants in zebrafish). While we recover a very small number of female crabp2a/b mutants, defects in the somatic gonad that effect sex differentiation appear to result in complete sex reversal, where only one sex is recovered. Our data support the hypothesis that reduced germ cell number in crabp2a/b mutants results in predominately male development and is consistent with the well-known impact of germ cell number on female sexual development in zebrafish.

References:
Matson CK et al (2011). DMRT1 prevents female reprogramming in the postnatal mammalian testis. Nature. 20;476(7358):101-4. doi:10.1038/nature10239.
Uhlenhaut NH et al. (2009). Somatic sex reprogramming of adult ovaries to testes by FOXL2 ablation. Cell. 139(6):1130-42. doi: 10.1016/j.cell.2009.
Webster KA et al. (2017). Dmrt1 is necessary for male sexual development in zebrafish. Dev Biol. 422(1):33-46. doi: 10.1016/j.ydbio.2016.12.008.
Romano S et al (2020). Loss of dmrt1 restores zebrafish female fates in the absence ofcyp19a1a but not rbpms2a/b. Development. 147(18):dev190942. doi: 10.1242/dev.190942.

Fig. 2 – The authors attempt to show that both RA signaling and Crabp2a are localized to germ cells. Better images are needed here to support this claim. The YFP reporter for RA signaling is very weak at both 12 and 23 dpf. At 13 dpf, the investigators use the antibody against Crabp2a to demonstrate that this gene is expressed in germ cells. This stain is also very weak and difficult to interpret.

In Figure 2, rare:YFP signal is the native fluorescence signal that was imaged post fixation. Hence, the weak signal. We have adjusted the contrast of the panels to highlight the signal and reduce the background fluorescence in Fig 2C (12 dpf). We have replaced the images for 23 dpf with another representative image which shows stronger YFP expression.

We have attempted Crabp2 antibody staining many times, with various methods for amplifying the signal, and unfortunately have been unable improve these results. We have adjusted the contrast of the image in panel 2K to help reduce the background fluorescence and highlight the signal. Our complementary HCR data in Figure S2 also support germ cell expression of crabp2, though it is difficult to rule out that there are very low levels in somatic cells.

Accordingly, we have added a line in the Results/Discussion (p. 12, line 241) at the end of the section on RA signaling stating “These results support a cell-autonomous role for crabp2s and RA signaling in GC development, though we cannot rule out some role in somatic cells.”

Fig. 3 -- Here the authors attempt to show that proliferation is reduced in germ cells using BrdU incorporation. However, I have a difficult time seeing BrdU labeling in germ cells of wild type gonads on 7dpf, whereas I see a major reduction in somatic cell proliferation in crabp2a mutants at this stage. At 12dpf, BrdU incorporation in wild type germ cell nuclei is more convincing, but I don’t see a big difference in mutant gonads.

© 2024. Published by The Company of Biologists under the terms of the Creative Commons Attribution License (https://creativecommons.org/licenses/by/4.0/).

We also attempted additional BrdU experiments multiple times. We have magnified insets and arrowheads that mark the germ cell nuclei showing localization of BrdU. We have adjusted the contrast of the BrdU panels to improve signal versus background. We have replaced panels for 12 dpf with better representative single plane images that clearly show specific GCs that with strong BrdU signal. We have also quantified these data in Figure 3R showing a modest though significant reduction in crabp2a/b mutants. These data are consistent with the reduced GC numbers that we quantify in the same figure.

In addition, pHH3 staining of gonads at 11 dpf in Suppl Fig 4 clearly show differences between WT and crabp2a/b mutants.

Fig. 4 – Addition of RA to culture medium does increase gonad size and BrdU incorporation, but again this seems to occur most obviously in nuclei surrounding germ cells. Higher magnification images of germ cell compared to somatic cell nuclei might alter this interpretation.

There is significant BrdU incorporation in somatic cells across all conditions and genotypes. This is expected at the stages examined. One would need to quantify the proportion of proliferative somatic cells to determine if the percentage of mitotic somatic cells increases following RA treatment. We have added insets showing magnified views of the GCs with arrowheads pointing to nuclei with BrdU signal.

Third decision letter

MS ID#: DEVELOP/2023/202549

MS TITLE: Cellular retinoic acid binding proteins regulate germ cell proliferation and sex determination in zebrafish

AUTHORS: Lianna Fung, Daniel B Dranow, Arul Subramanian, Natalia Libby, and Thomas F Schilling

Thanks for making the additional revisions to your manuscript that we are now happy to publish. I am happy for you to add the additional figure to the main paper and so please resubmit files with that figure included. At the editing stage, our executive editor (Katherine Brown) will decide whether to publish as a short report with an extra figure or a short article.

Third revision

Author response to reviewers' comments

There are no comments from reviewers to address at this round of revision.

Fourth decision letter

MS ID#: DEVELOP/2023/202549

MS TITLE: Cellular retinoic acid binding proteins regulate germ cell proliferation and sex determination in zebrafish

AUTHORS: Lianna Fung, Daniel B Dranow, Arul Subramanian, Natalia Libby, and Thomas F Schilling

I am happy to tell you that your manuscript has been accepted for publication in Development, pending our standard publication integrity checks.

© 2024. Published by The Company of Biologists under the terms of the Creative Commons Attribution License (https://creativecommons.org/licenses/by/4.0/).
